# Regenerative capacity of trophoblast stem cell-derived extracellular vesicles on mesenchymal stem cells

**DOI:** 10.1186/s40824-023-00396-5

**Published:** 2023-06-27

**Authors:** Yoon-Young Go, Chan-mi Lee, Sung-Won Chae, Jae-Jun Song

**Affiliations:** 1 Department of Otorhinolaryngology-Head and Neck Surgery, Korea University Guro Hospital, 80 Guro-Dong, Guro-Gu, Seoul, 08308 South Korea; 2 Institute for Health Care Convergence Center, Korea University Guro Hospital, Seoul, 08308 Republic of Korea

**Keywords:** Trophoblast stem cells, Extracellular vesicles, Mesenchymal stem cells, Regenerative properties, NGF, Akt pathway

## Abstract

**Background:**

Human mesenchymal stem cells (MSCs) are therapeutic for clinical applications because of their excellent immunomodulatory and multiple lineage differentiation abilities at tissue injury sites. However, insufficient number of cells and lack of regenerative properties during in vitro expansion still limit the clinical applicability of MSC therapies. Here, we demonstrated a preconditioning strategy with trophoblast stem cell-derived extracellular vesicles (TSC-EVs) to boost the proliferation and regenerative capacity of MSCs.

**Methods:**

We employed cell proliferation analyses such as CCK8 and BrdU assays to determine the proliferation-promoting role of TSC-EVs on MSCs. Osteogenic effects of TSC-EVs on MSCs were assessed by alkaline phosphatase (ALP) activity, calcium assays, and calvarial bone defect animal models. For skin regenerative effects, skin wound mice model was exploited to analyze wound-healing rate in this study, as well as immunofluorescence and histological staining evaluates. We also performed the small RNA profiling and RNA-sequencing analyzes to understand the cellular mechanism of TSC-EVs on MSCs.

**Results:**

TSC-EVs significantly promoted MSC proliferation under xeno-free conditions and facilitated the therapeutic effects of MSCs, including osteogenesis, anti-senescence, and wound healing. Transcriptomic analysis also provided evidence that specific microRNAs in TSC-EVs and differentially expressed genes (DEGs) in TSC-EV-treated MSCs showed the possibility of TSC-EVs triggering the regenerative abilities of MSCs with cytokine interaction. Hence, we found that NGF/Akt signaling mediated the regenerative effects of TSC-EVs on MSCs as a particular cellular signaling pathway.

**Conclusion:**

The results of this study demonstrated the functional properties of TSC-EVs on MSCs for MSC-based therapeutic applications, suggesting that TSC-EVs may serve as a potential preconditioning source for MSC therapy in the clinical field of regenerative medicine.

**Graphical abstract:**

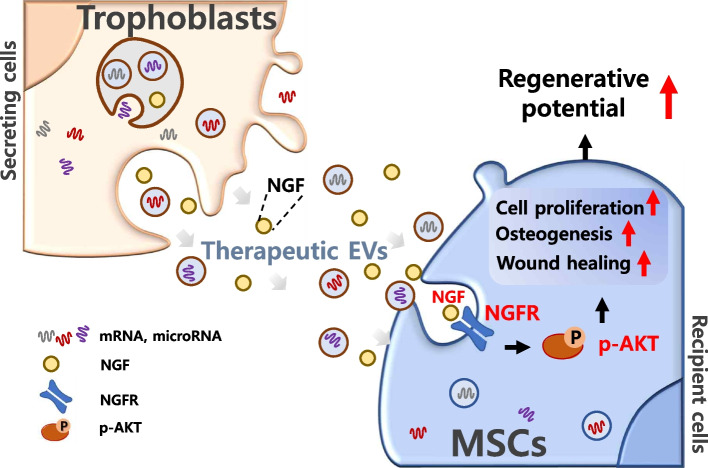

**Supplementary Information:**

The online version contains supplementary material available at 10.1186/s40824-023-00396-5.

## Introduction

MSCs have limited pluripotency and capacity for cell differentiation compared to embryonic stem cells (ESCs) and induced pluripotent stem cells (iPSCs). However, they are easy to obtain from human adult tissues without ethical issues and play an essential role in tissue repair and organ homeostasis [1, 2]. Following the expansion of stem cell therapy using autologous MSC for tissue regeneration [3]. The population of MSC in human tissues is insufficient, and obtaining a high cellular yield and maintaining biological capacity during in vitro expansion remains challenging [4]. Furthermore, conventional MSC expansion with fetal bovine serum (FBS) causes immune- and inflammation-related problems following the exposure of xenogeneic pathogens from FBS [5]. This necessitates the discovery of a better method with xeno-free conditions to improve MSC potency in the clinical field of regenerative medicine. Many researchers have attempted to upgrade the properties of naïve MSCs using genetic engineering or preconditioning method [6–8], a genetic modification approach was that overexpress CXCL4/CXCL7 encoding genes to serve promoting the MSCs proliferation and migration abilities [9]. In addition, different concentrations of oxygen during MSC in vitro expansion are related to several MSC properties; for instance, 1% oxygen promotes proliferation, stemness, and anti-senescence features, while 2% oxygen increases cytokine expression levels to enhance the angiogenesis of MSCs [10, 11].

Trophoblasts are multifunctional placental cells critical in pregnancy maintenance and communication between the fetus and mother [12]. After implantation, self-renewal and proliferative cytotrophoblasts appear in the early first trimester (6-9 weeks) and can help identify trophoblast stem cells (TSCs) [13]. They have been widely studied in obstetrics and gynecology because abnormal trophoblast differentiation can cause several placenta-related disorders during pregnancy [14, 15]. In 2014, Salomon et al. were the first to assess nanoparticle-like exosomes released from trophoblasts and determine the functional activity of trophoblast exosomes in human uterine spiral artery remodeling by inducing vascular smooth muscle cell migration for successful pregnancy [16]. A recent study supports the pivotal role of trophoblast exosomes in that extracellular vesicles (EVs) derived from TSCs promote fertility by regulating endometrial cell receptivity [17]. Moreover, microRNAs in TSC-EVs regulate the processes of embryo implantation and the uterine environment, such as inflammation and angiogenesis [18]. Thus, TSC-EVs have a regulatory ability to change the morphology and physiological state of cells, consequently leading to tissue remodeling. However, TSC-EV applications in tissue engineering and regenerative medicine have received little attention.

Herein, we explored the effects of TSC-EVs as a preconditioning source on MSC properties. We also determined the role of the NGF/AKT pathway in the regenerative effect of MSCs via TSC-EV treatment.

## Materials and methods

### Preparation of serum-free CM and EVs

TSCs and MSCs were maintained in a growth medium with exosome depleted FBS (Gibco, Grand Island, NY, USA) until 60–70% confluence. The cells were then incubated in growth medium without FBS for 24 h after three washes with PBS. CM collected from TSCs and MSCs was centrifuged at 300 × *g* for 10 min and then followed second centrifugation at 2,000 × *g* for 10 min to discard the cell debris. The serum-free CM of each stem cell was prepared for the experiment by filtering using a 0.22-µm pore filter (syringe filter; Corning, NY, USA). EVs were isolated from serum-free CM using the ultracentrifuge method with differential centrifugation steps as previously reported [19]. Briefly, CM was ultracentrifuged at 10,000 × *g* for 30 min and then serially followed by two ultra-centrifugations at 100,000 × *g* for 70 min to purify the EVs. EVs were prepared by resuspension in PBS. The particle size and concentration of EVs were measured by nanoparticle tracking analysis (NTA). The morphology and expression of exosome marker proteins of EVs were determined using transmission electron microscopy (TEM) and western blotting, respectively. The detailed methods for NTA and TEM can be found in the Supplemental Methods section.

### Cell culture

Human TSCs were obtained from American Type Culture Collection (ATCC) (HTR8-SVneo, CRL-3271; VA, USA) and cultured in RPMI-1640 (Gibco) supplemented with 5% FBS (Gibco) and 1% penicillin/streptomycin (10,000 U/mL) (Gibco) at 37 °C with 5% CO_2_. The human MSCs used in this study, including bone marrow-derived MSCs (BM-MSCs), umbilical cord-derived MSCs (UC-MSCs), and adipose tissue-derived MSCs (AD-MSCs), were purchased from PromoCell (PromoCell; Heidelberg, Germany). All cells were grown in MEM (Gibco) medium supplemented with 5% FBS and 1% penicillin/streptomycin (10,000 U/mL) (Gibco) was used for cell culture. Serum-free medium (SFM) indicates FBS-excluded growth medium, which does not contain animal-derived materials. All cells were maintained in a humidified atmosphere at 37 °C with 5% CO_2._

### Cell proliferation assay

MSCs (1 × 10^4^ cells/well) were seeded into 96-well plates with SFM in the presence or absence of TSC-CM or EVs for 24 and 48 h, respectively. Cell Counting Kit (CCK) 8 (Dojindo Laboratories, Kumamoto, Japan) solution was added to each well and then incubated for 2 h at 37 °C. The optical density of the live MSCs was measured at 450 nm using a microplate spectrometer. For bromodeoxyuridine (BrdU) staining, MSCs were cultured in the presence or absence of TSC-CM or EVs for 24 h in SFM, and BrdU (Sigma) was added to the cells before the end of the culture. The cells were then incubated with anti-BrdU antibody (Invitrogen) for 20 h at 4 °C, followed by immunofluorescence staining. Proliferative cells were determined by the ratio of fluorescence-positive cells in three different fields of images. MSCs were pretreated with anti-nerve growth factor (NGF) (R&D system, Minneapolis, MN, USA) for 1 h followed by treatment with TSC-EVs.

### Small RNA-sequencing analysis

Total RNA was extracted from exosomes using the mirVana microRNA isolation kit (Life Technologies, MD, USA), and small RNA profiling was performed on the Illumina platform (Illumina, CA, USA) in accordance with the SMARTer smRNA-Seq Kit (Takara Bio USA, CA, USA) for the Illumina user manual. The exosomal RNA derived from TSCs and MSCs (200 ng) was subjected to polyadenylation, cDNA synthesis, and polymerase chain reaction (PCR) amplification (16 cycles). Raw sequence reads were filtered to remove rRNA and adapter sequences. The trimmed reads were aligned with the reference genome using miRNBase v22.1, STAR, and RNAcentral 14.0, for classification [20–22]. Bowtie aligner and miRDeep2 were used to predict novel microRNAs or to quantify the read count of microRNAs [23, 24]. Differentially expressed microRNAs were identified by comparing the read count values of microRNAs using statistical methods, such as fold-change (> twofold) and *p-value* (< 0.05). The mirDIP database was used to predict the target genes of microRNAs [25]. The selected microRNA target genes with high stringency were presented in at least five databases and were above the top two-thirds in confidence, as curated by miRDIP.

### RNA-sequencing

Total RNA was isolated from control MSCs and TSC-EV-treated MSCs for 48 h. DNA contamination in Total RNA was removed using DNase, and RNA was quantified using the Quant-iT RiboGreen assay (Invitrogen). Transcriptomic study of MSCs, either TSC-EV-treated MSCs or control MSCs, was performed using the TruSeq Stranded mRNA Library Prep kit, as previously reported [26]. Briefly, the TruSeq Stranded mRNA Reference Guide (Illumina, part number 1000000040498v00, San Diego, CA, USA) was used to construct a transcriptome library according to the manufacturer’s protocol. Fragmented RNA (1 μg) was reverse transcribed to generate cDNA and different adapters were attached to both ends of the cDNA fragment and ligated. The final cDNA (200-400 bp) was isolated after PCR amplification using the KAPA Library Quantification kits (Kapa Biosystems, MA, USA), and paired-end sequencing was performed. Low-quality sequences, such as adapter sequences, contaminant DNA, and PCR duplicates were removed by mapping the reference genome of *Homo sapiens* (GRCh38) sequences using the HISAT2 program (v2.1.0) [27]. After quality control using HISAT2, aligned reads were assembled using the StringTie2 program (v2.1.3b) to quantify and normalize the relative expression levels of genes by considering read count, transcript length, and depth of coverage [28]. Statistical analysis using Benjamini, and Hochberg algorithm (*p* < 0.05) was performed to determine the differentially expressed genes (DEGs) between the two groups. DEGs were further investigated for functional biological processes and pathways via bioinformatic analysis. Multidimensional Scaling (MDS) plots were generated from normalized log values of genes in each group that expressed different variabilities of the two components (MDS 1 and 2) to evaluate the similarity among biological replicates.

### Bioinformatic analysis

The Database for Annotation, Visualization, and Integrated Discovery (DAVID) and Kyoto Encyclopedia of Genes and Genomes (KEGG) were used to determine the gene ontology (GO) and enriched biological pathways for significant genes [29, 30]. Gene Set Enrichment Analysis (GSEA)/Molecular Signatures Database (MSigDB) was used to statistically define the set of genes between control MSCs and TSC-EV-treated MSCs based on a significant enrichment score using the false discovery rate (FDR) [31].

The miRNA target genes were further analyzed using the DAVID and PANTHER tools to identify enriched biological functions using GO annotations and pathway analysis [32]. Only GO terms and pathways whose expression showed more than two-fold change and statistical implications (*p* < 0.05) were regarded as exerting biological influences. The Benjamini and Hochberg method using the FDR threshold (*p* < 0.05) was used to calculate the adjusted *p-value*. ClueGO was used to visualize the enriched biological processes and pathways of the miRNA target genes [33]. Pathway analysis of functional protein–protein interactions was conducted using the STRING database [34]. The results showed a full string network that applied the species limited to “Homo sapiens” with high confidence (0.7) and statistical significance (FDR-adjusted *p* < 0.05). Cytoscape software version 3.9.0 was used to visualize the ClueGO and STRING networks [35]. The steps of transcriptomic studies are shown by a flowchart (Figure S1A and B).

### Osteogenic differentiation of MSCs

Bone marrow-derived human MSCs (1 × 10^5^ cells/well) were seeded in 24-well plates with growth medium and cultured until 95% confluency. The growth medium was replaced with osteogenic induction medium (OIM). OIM contains additionally 10 nM dexamethasone (Sigma), 0.2 mM ascorbic acid (Sigma), and 10 mM β-glycerol phosphate (Sigma) in the growth medium. Fresh OIM was replaced every 2-3 days during culture. The activity of alkaline phosphatase (ALP) was determined using a SensoLyte *p*NPP Alkaline Phosphatase Assay Kit (ANASPEC, Fremont, CA, USA) according to the manufacturer’s protocol. Briefly, cells were washed and lysed with a lysis solution containing 0.5% Triton. *p*NPP ALP was added to the samples, and the color changes were measured at 405 nm wavelength using a microplate reader. The calcium deposition on cells was decalcified by 0.6 N HCl and determined using Quanti Chrom Calcium Assay kit (DICA-500; BioAssay Systems, Hayward, CA, USA) according to the manufacturer’s instructions. The color changes according to different calcium contents were evaluated using a microplate reader with 612 nm absorbance. To determine the rate of mineralization, the cells were stained with Alizarin Red S solution (Millipore, Darmstadt, Germany) for 15 min after fixation in 4% paraformaldehyde for 10 min. Red color-stained cells were imaged and de-stained with 100 mM cetylpyridinium chloride (Sigma), and the different color changes were quantified using a microplate reader at 570 nm.

### A rat calvaria defect model

All animals were housed in a standard pathogen-free facility at the animal research center of Korea University Guro Hospital and followed the guidelines of the Institutional Animal Care and Use Committee (IACUC) for animal ethics and welfare. The experimental protocols for animal experiments were approved by the IACUC of Korea University (KOREA-2020-0030). For the critical bone-defect model in rat calvaria, 8-week-old Sprague-Dawley rats were used. A circular (6-mm diameter) critical defect was created in the parietal skull bones using a trephine bur. The excised calvaria bone was removed, and the precultured MSCs (1 × 10^6^ cells/scaffold) in the Protinet scaffold (Danagreen, Seoul, Korea) with or without TSC-EVs for 5 days were immediately implanted in the defect region. The defect-only group was used as the control group. The surgical area of the calvarial bones was sutured and bone regeneration was determined after 8 weeks. A microcomputed tomography (micro-CT) instrument (SKYSCAN 1176, Bruker; resolution of 9-μm pixel/0.5 mm AI) was used to evaluate the bone formation of defective sites before histological study.

### Histological analysis

For histochemical analysis, skull bone was decalcified in 10% EDTA (pH 7.4) for 14 days after fixation in 10% formalin. Tissue samples were then embedded in paraffin and sliced at 5-μm thickness using a rotary microtome (RM2255, Leica). After deparaffinization and dehydration of the tissue sections, hematoxylin and eosin (H&E) and Masson’s trichrome (MT) staining were performed. For H&E staining, staining with hematoxylin solution (Sigma) for 5 min and washing with tap water were performed, immediately soaked in an Eosin-Y solution (Sigma) for 1 min, and washed with tap water. For MT staining, the tissue sections were immersed in hematoxylin solution for 10 min, serially followed the steps by washing with acetic acid (1%) (Sigma), incubation in acid orange G solution (Sigma), and final staining with light blue for 5 min. The stained images were obtained using an H-filter in color mode.

### Senescence associated beta-galactosidase (SA β-gal) assay

MSCs (1 × 10^6^ cells/well) were seeded into 6-well plates with SFM and incubated in the presence or absence of TSC secretomes for 24 h. The cells were then stained with SA β-gal solution (Sigma) after fixation. Three different images were taken from each group, and the high, low, and negatively stained cells were quantified.

### Wound-healing assay

The in vivo wound-healing assay was performed as previously described [36]. Briefly, BALB/c mice were anesthetized, the skin on the dorsal surface was excised under the panniculus carnosus muscle, and a full-thickness wound (10 mm diameter) was created. MSCs (1 × 10^6^ cells) were immediately implanted into the injured area with TSC-CM, MSC-CM, TSC-EVs, MSC-EVs, or TSC-EVs + anti-NGF. Anti-NGF was pretreated to the wound area with MSCs for 1 h, and then TSC-EVs were added to the injury site. The wound area was measured every 3 days until day 15 and calculated using a previously reported formula [37]: % of wound area = (total wound area – present wound area)/total wound area × 100.

### Quantitative real-time PCR

Total RNA (1 μg) was converted to cDNA (20 μL) using the PrimeScrip first-strand cDNA Synthesis Kit (Takara Bio, Tokyo, Japan), and real-time (RT)-PCR) was performed using the ABI Prism 7500 Detection System (Applied Biosystems, CA, USA) according to the manufacturer’s protocol. Gene expression levels were normalized to that of GAPDH, and relative gene expression was analyzed using the 2 ^(−∆∆Ct)^ method [38]. Specific primer sequences are listed in Supplementary Table 1.

To confirm the miRNA sequencing results, miR-1246 (probe info; 483023_mir) (Thermo Fisher Scientific, MA, USA), miR-335-5p (probe info; 478324_mir), miR-144-3p (probe info; 477913_mir), and miR-150-5p (probe info; 477918_mir) were quantified as exosomal miRNAs derived from TSC-EVs and MSC-EVs. cDNA was synthesized using a Superscript II RT-PCR System (Invitrogen). Subsequently, qRT-PCR was performed using an ABI PRISM 7900HT sequence detection system with a Universal Master mix (Applied Biosystems). All PCR were performed in triplicate, and the data were analyzed using Sequence Detector software (Applied Biosystems). Cel-miR-3p (478291_mir) was used as a control for the relative quantification of miRNAs, and the data were normalized and compared using the 2 ^(−∆∆Ct)^ method.

### Western blotting and immunofluorescence staining

For western blotting, cells and EVs were lysed using RIPA buffer (Invitrogen) with protease and phosphatase inhibitors (Roche, Indianapolis, IN, USA), and protein concentrations in each sample were calculated using Quick Star Bradford dye reagent (Bio-Rad, Hercules, CA, USA). Equal amounts of protein samples were subjected to sodium dodecyl sulfate-polyacrylamide gel electrophoresis and transferred to a PVDF membrane (Bio-Rad). The membranes were blocked with 5% (w/v) skim milk and 0.1% Tween-20 in Tris based buffer for 30 min and then incubated with primary antibodies for 20 h at 4 °C. The membranes were probed with horseradish peroxidase-conjugated secondary antibodies for 1 h at room temperature, and the protein expression bands were observed using chemiluminescence (Bio-Rad). Full-length original blots are included in the Supplementary Information.

For immunohistochemical staining, the rehydrated sections or cultured cells were permeabilized with 0.4% Triton for 5 min. After blocking with 0.3% bovine serum albumin (Sigma) for 30 min, samples were incubated with primary antibodies for 2 h at 4 °C and applied in fluorescence-conjugated secondary antibodies for 1 h in the dark. Finally, the samples were mounted using Fluoroshield with DAPI (Sigma), and immunofluorescence images were captured using a Zeiss LSM 700 confocal microscope. The antibodies used in this study are listed in Supplementary Table 2.

### Statistical analysis

All quantitative experiments were performed using triplicate samples, and the data are presented as the mean ± standard deviation. Student’s t-*t*est and one-way ANOVA with post-hoc Tukey’s tests were used to determine statistical differences among the experimental groups. Statistical significance was considered at *p < 0.05, **p < 0.01, and ***p < 0.001.

## Results

### TSC-CM and -EVs induce proliferation ability of MSCs

To determine the potential of the TSC-derived secretomes to sense MSCs proliferation, TSC-derived conditioned medium (TSC-CM) and extracellular vesicles (TSC-EVs) were isolated using a serial ultracentrifugation method (Figure S2A). EV surrounded by a lipid bilayer with 100-150 an average diameter was observed by TEM and NTA (Figure S2B, C). We determined the specific exosome marker proteins (ALIX, CD63, and CD81) and yield of EVs from TSCs and MSCs (Figure S2D, E).

**Fig. 1:**
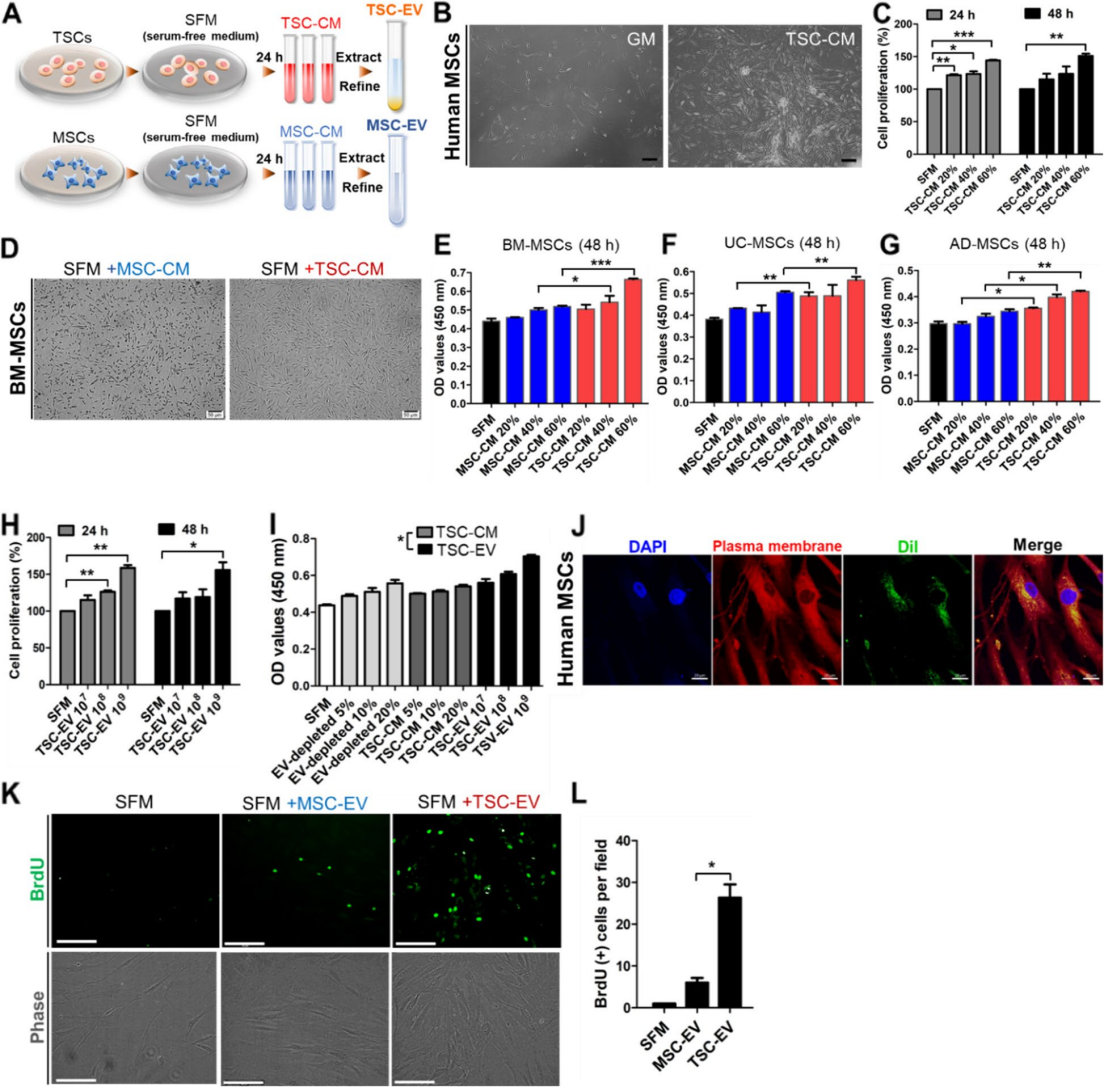
Promoting effect of TSC-derived secretome on MSC proliferation in SFM. **A** Scheme of CM and EV collected from TSC and MSC. **B** Morphological changes in TSC-CM treated MSCs during growth. Scale bars, 50 μm. **C** Dose-dependent TSC-CM treated to MSCs in SFM; then, the percentage of cell proliferation was determined at 24 h and 48 h by CCK8. **D** Different morphology and (**E**) growth rate of BM-MSCs on MSC-CM and TSC-CM treated MSCs in SFM, respectively. Scale bars, 50 μm. **F** UC-MSCs and (**G**) AD-MSCs were cultured with MSC-CM or TSC-CM for 48 h in SFM. The proliferation rate was analyzed by CCK8. **H** Determination of cell proliferation for dose-dependent TSC-EV treated to MSCs in SFM was performed by CCK8. **I** The rate of cell proliferation was compared in MSCs treated with TSC-CM, EV-depleted TSC-CM, and TSC-EV. **J** Internalization of Dil-labeled TSC-EV (green) onto MSCs was evaluated by immunofluorescence detection. DAPI, blue; plasma membrane, red. Scale bars, 10 μm. **K** Representative images of BrdU-positive cells (green) in MSC cultured with TSC-EV for 24 h in SFM. Scale bars, 125 μm. **L** BrdU-positive cells were counted. MSCs culture with SFM-only was used as control. Error bars presented the mean and SD. (*n* = 3; *p < 0.05, **p < 0.01, and ***p < 0.001)

MSCs are well-known multipotent stem cells that can be applied in clinical fields, but it is necessary to expand the cells to xeno-free conditions before use. To address this, TSC-CM, -EVs, and MSC-CM, -EVs were first prepared in SFM to maintain the xeno-free culture of MSCs (Fig. 1A). MSC-CM and -EVs were used as the positive control in this study. BM-MSCs were cultured in TSC-CM at various concentrations for 24–72 h, after which the proliferation efficacy of MSCs was determined. High accumulation of MSCs was detected (Fig. 1B, S3A), and the analysis of cell proliferation showed that the addition of TSC-CM significantly induced the growth potential of MSCs in a time- and dose-dependent manner (Figure S3B). The rate of cell proliferation in cultured MSCs with SFM was significantly decreased compared to that in FBS-containing growth medium (GM) (Figure S3C), but TSC-CM treatment overcame the harsh condition of MSCs culture without animal supplementation and significantly increased the proliferation of MSCs (Fig. 1C). Compared to the TSC-CM treatment, MSC-CM did not provide sufficient conditions for MSCs cultured in SFM for cell proliferation (Fig. 1D). TSC-CM also stimulated the proliferation rate of UC-MSCs and AD-MSCs as well as BM-MSCs under SFM conditions (Fig. 1E, F, G). Furthermore, TSC-EVs also enhanced the proliferation potential of MSCs cultured in SFM, as analyzed by the CCK assay (Fig. 1H). We examined the proliferative ability of TSC-CM and TSC-EVs and found that TSC-EV treatment significantly increased MSCs proliferation compared with TSC-CM (Fig. 1I). This might indicate that TSC-EVs compactly possess the active biological components of the TSC-derived secretome. TSC-EV internalization by MSCs was detected after incubation between Dil-labeled TSC-EVs and MSCs in vitro (Fig. 1J). The number of BrdU-positive cells in SFM-cultured MSCs treated with TSC-EVs was markedly increased compared to those in the SFM control medium, TSC-CM-, and MSC-EV-treated groups (Fig. 1K, L and S3D, E). Overall, these results indicate that TSC-CM and -EV treatment provided a suitable environment for xeno-free MSCs cultivation.

### MicroRNAs in TSC-EVs exhibit regenerative capacity

**Fig. 2:**
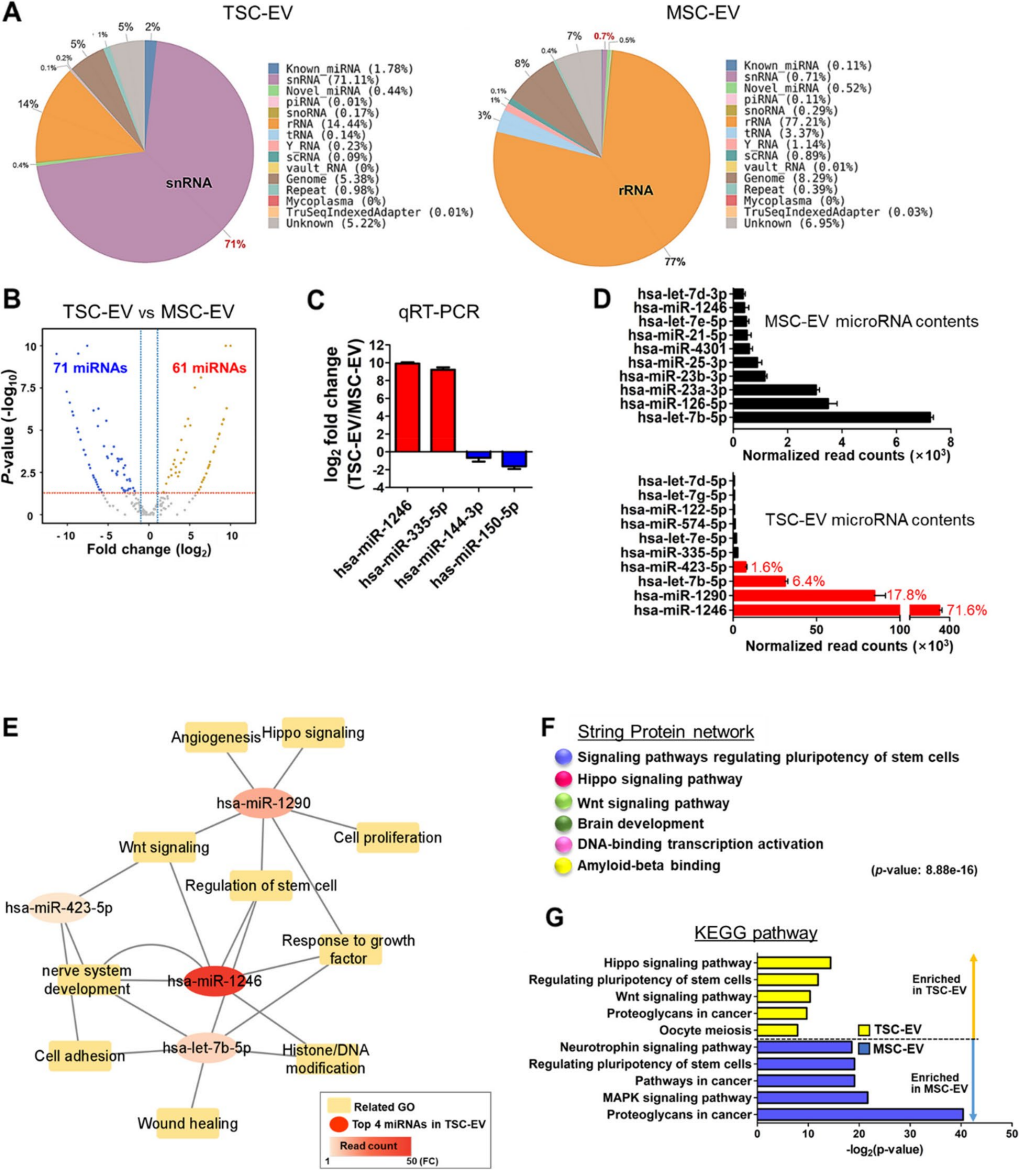
Exploring possible bioactivity of TSC-EVs in MicroRNA profiling (**A**) Venn diagram comparing the different compositions of small RNAs identified in TSC- and MSC-EV. **B** Differentially expressed microRNAs in TSC-EV compared with MSC-EV were represented by the volcano plot based on fold-change (FC) and *p* values (|FC|≥ 1.5 adjusted with *p* < 0.05). **C** Up- or downregulated microRNAs in TSC-EV verified the microRNA profiling results via quantitative RT-PCR. **D** Top 10 microRNAs in each stem cells-derived EV were presented by graph. The most abundant top 4 microRNAs in TSC-EV were indicated by the red color. **E** Enrichment map of biological effects targeted by the four microRNAs overexpressed in TSC-EV. The connection nodes indicate significantly enriched biological processes and pathways (*p* < 0.05). **F** STRING analysis presented six functional clusters, stem cell regulation, Hippo signaling, Wnt signaling, brain development, DNA-binding transcription activation, and amyloid-beta binding, regulated by predicted targets of four overexpressed microRNAs in TSC-EV. **G** DAVID analysis revealed differential changes in enriched KEGG pathway caused by four microRNAs overexpressed in TSC-EV, compared with KEGG pathway of top 1% microRNAs expressed in MSC-EV (enriched in TSC-EV, yellow; enriched in MSC-EV, blue)

MicroRNA-containing EVs can modulate gene expression by regulating target mRNAs during the post-transcriptional process, thereby influencing cell fate decisions, as well as overall tissue remodeling and regeneration [39, 40]. Moreover, the composition and number of small RNAs including microRNAs in exosomes vary depending on cell type and cellular status, which can exert various biological effects [41]. Therefore, small RNA-sequencing was performed to investigate the functional properties of TSC-EVs in cellular processes and compare the microRNA profiles of EVs derived from both TSCs and MSCs. Raw sequencing reads were trimmed, and 504,465 (2.08%) and 44,348 (0.51%) microRNA sequences for TSC and MSC, respectively, were identified (Table S3). Figure 2A shows the differential RNA composition between TSC- and MSC-EVs; TSC-EVs contained 71% small nuclear RNA (snRNA), while MSC-EVs contained 77% ribosomal RNA (rRNA).

The differentially expressed microRNAs in TSC-EVs compared to the microRNAs expressed in MSC-EVs were determined. The 132 microRNAs (UP:61, Down:71) were differentially expressed in TSC-EVs (Fig. 2B), and the sequencing results were confirmed using quantitative RT-PCR (Fig. 2C, S4A). We found different microRNA compositions of these two types of stem cell-derived EVs from the list of identified microRNAs in each EV (Fig. 2D); the top four microRNAs in TSC-EVs, miR-1246, miR-1290, let-7b-5p, and miR-423-5p, accounted for 97.5% of all EVs microRNAs; they were mostly found in TSC-EVs. Numerous other microRNAs were found in TSC-EVs, but the remaining 93 miRNAs had a very small proportion of total reads (0.01% to 0.56%) (Supplemental Information – Excel Table 1), which is unlikely to exert significant biological effects on cells. Thus, we selected the top four microRNAs most abundantly expressed in TSC-EVs and used DAVID to examine the relevance of each microRNA and its biological function. The targets of overexpressed miR-1246 were linked to nervous system development, growth factor response, histone/DNA modification, stem cell regulation, and the Wnt signaling pathway (Fig. 2E). Other biological processes and pathways related to angiogenesis, cell proliferation, cell adhesion, wound healing, and Hippo signaling were also significantly enriched. STRING and ClueGo analysis consistently visualized the protein networks using functional enrichment of targets predicted from the majority of four TSC-EV microRNAs (Fig. 2F, S4B, and C). The GO terms and KEGG pathways were analyzed to compare the significant biological influences of these top microRNA (read counts greater than 1%) target genes between TSC-EVs and MSC-EVs, indicating that TSC- and MSC-EVs have distinct biological effects that are triggered by different microRNA compositions (Fig. 2G, S4D-G). Analysis of the microRNA landscape of TSC-EVs suggests that microRNAs in TSC-EVs have different biological properties than MSC-EVs and can exert regenerative effects by regulating the expression of their target genes.

### Transcriptome analysis of possible regeneration properties of TSC-EVs on MSCs

**Fig. 3:**
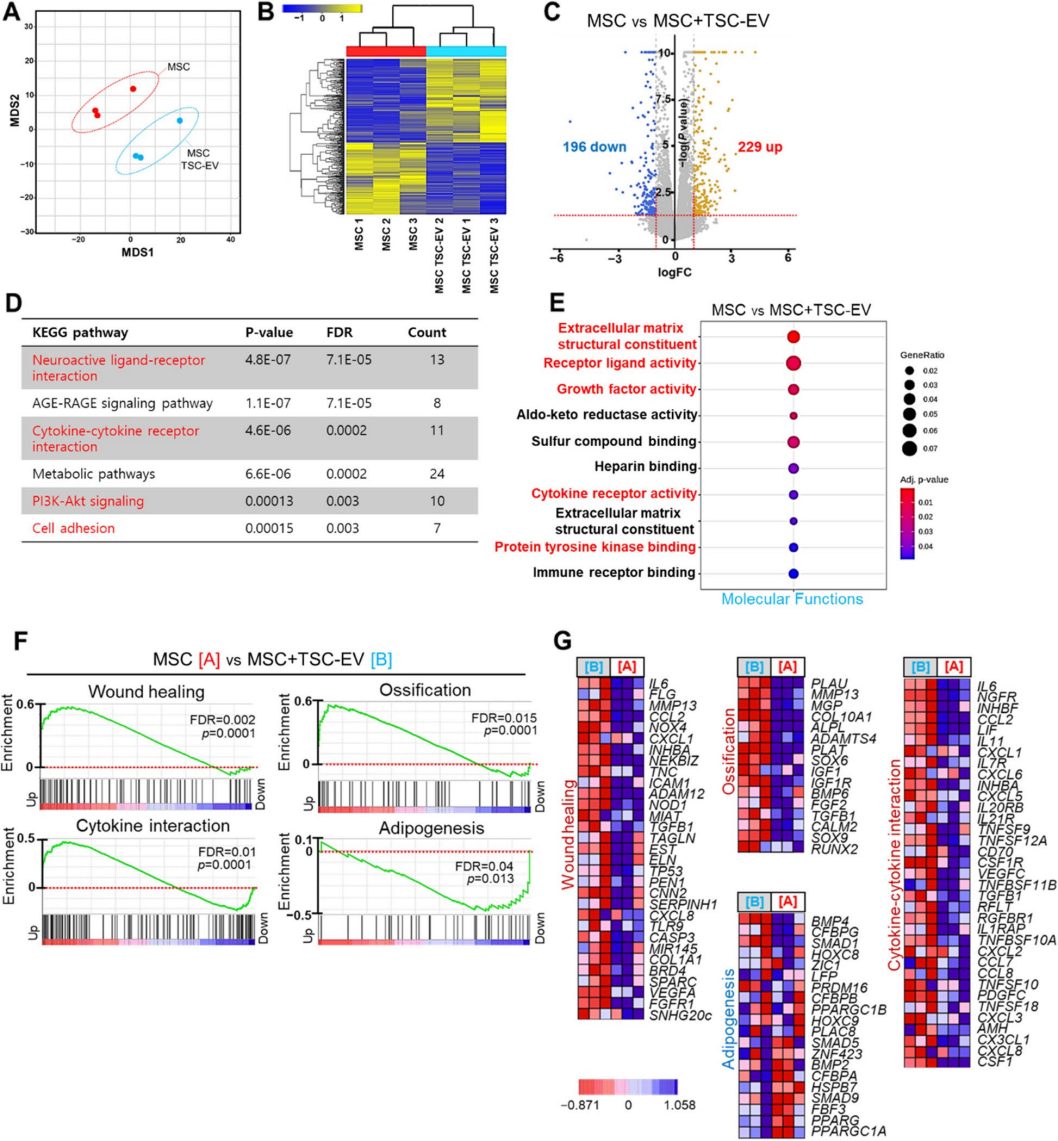
Transcriptomic analysis of TSC-EV-induced regenerative capacity on MSCs. **A** MDS plot of RNA-seq result represents the distinct cluster correspond to the differences in differentially expressed genes (DEG) between MSCs (red) and TSC-EV-treated MSCs (blue). The three replicates in each group show the close distance. **B** The heatmap clustering data visualize the change in differential gene expression levels across TSC-EV-treated MSCs compared with untreated control MSCs values (|FC|≥ 1.5 adjusted with *p* < 0.05). **C** The Volcano plot indicates differentially expressed up- and downregulated genes in response to TSC-EV treatment on MSCs. The numbers of significantly up- and downregulated genes are denoted. **D** Significant KEGG pathways enriched in TSC-EV-treated MSCs are showed by table with the statistical scores (FDR and *p*-value) and the number of significant genes. **E** The enriched molecular function in TSC-EV-treated MSCs is showed with adjusted p-value and gene expression ratio. **F** Gene set enrichment analysis (GSEA) in MSCs [A] and TSC-EV-treated MSCs [B] were subjected with enrichment score based on significance. Regeneration activity of MSCs related four enrichment plots are presented with up- or downregulated genes in each data set. **G** Heatmaps present DEG levels enriched to core biological processes or pathways in (**F**). DEGs associated with ossification, wound healing, and cytokine regulation were preferentially expressed in TSC-EV-treated MSCs gene expression profile. Negative correlation of Adipogenesis DEGs was analyzed in TSC-EV-treated MSCs enriched genes

We also performed RNA-sequencing (RNA-seq) analysis to better understand the regenerative capacities of TSC-EVs to improve the therapeutic functions of MSCs. The results of MDS and heat map analysis showed that TSC-EV treatment activated DEGs with statistical significance (Fig. 3A, B). TSC-EV-treated MSCs differentially expressed 425 genes, of which 229 genes were upregulated and 196 genes were downregulated (Fig. 3C). KEGG pathway analysis based on DEGs revealed that TSC-EVs activated neuroactive ligand-receptor interactions (e.g., *Npy4r*, *Cckar*), cytokine receptor interactions (e.g., *Ngfr*, *IL6*, *Col4a3*), and PI3K-Akt signaling (e.g., *Ngfr*, *IL6*, *Ccl2*, *Lif*) (Fig. 3D and S5A-C). Moreover, three GO categories were enriched in extracellular matrix organization that were involved in the structural changes of the MSCs microenvironment via TSC-EV treatment (Fig. 3E, S5D, E). The GSEA analysis indicated significantly up- and downregulated biological processes in TSC-EV-treated MSCs, positively related to ossification, cytokine interaction, wound healing, and negative regulation of adipogenesis (Fig. 3F, G). These results indicate that TSC-EVs possibly activated cytokine interaction related pathway in MSCs such as NGFR and Akt signaling, which could trigger the regenerative activities of MSCs in bone regeneration and wound healing processes.

### TSC-EVs promote bone regenerative efficacy of MSCs

**Fig. 4:**
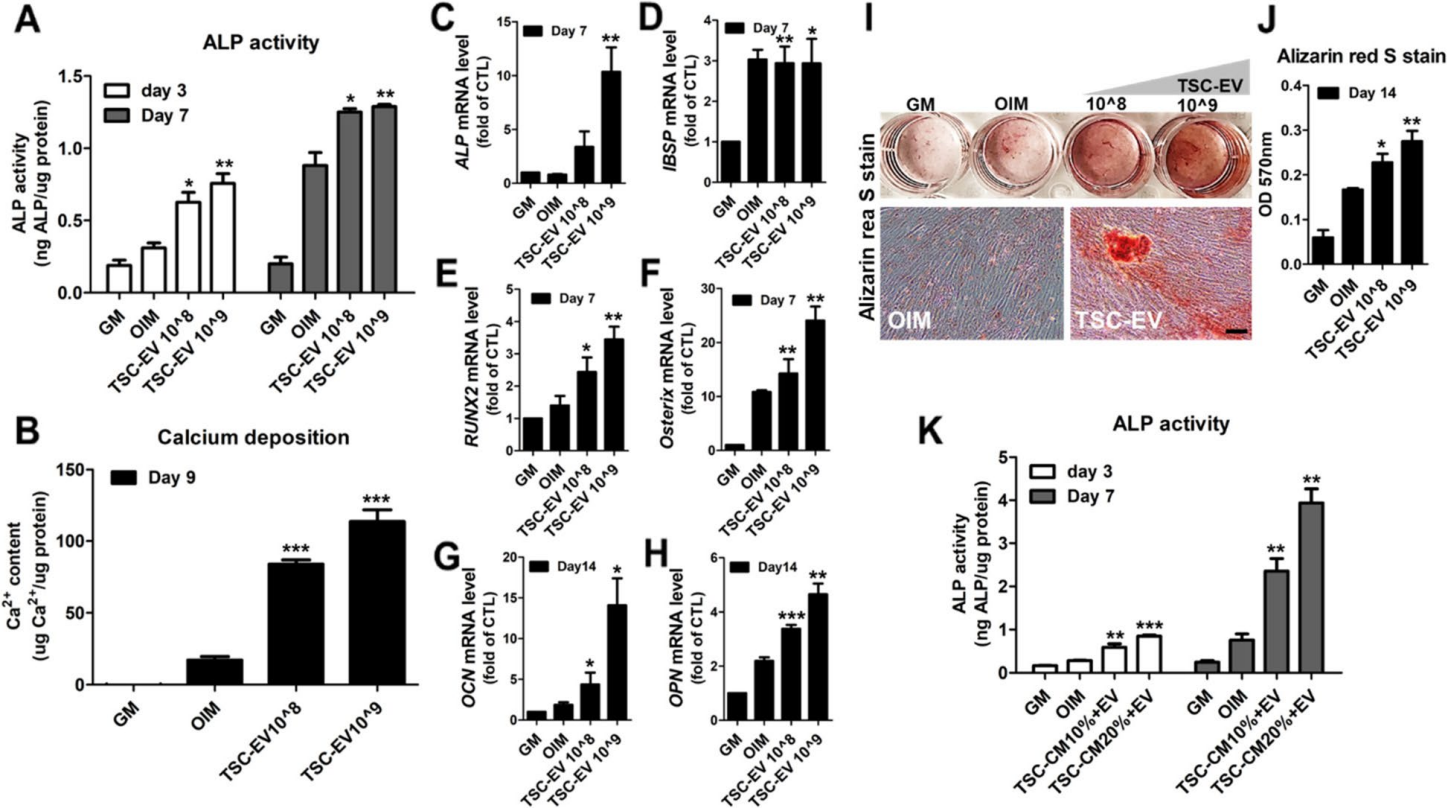
TSC-EVs enhanced osteogenic efficacy of MSCs. The ALP activity (**A**) and calcium content (**B**) of TSC-EV-treated MSCs were examined on the indicated day. **C**-**H** Relative expression levels of osteogenic marker genes in TSC-EV-treated MSCs were determined using quantitative RT-PCR. MSCs cultured with GM were used as controls. Representative image (**I**) and quantified staining (**J**) of Alizarin Red S-stained MSCs on day 14 in TSC-EV-treated MSCs compared to OIM. Scale bars, 100 μm. **K** The synergistic increase in ALP activity in TSC-EV-treated MSCs with additional TSC-CM was analyzed. Error bars represent the mean and SD. (*n* = 3; *p < 0.05, **p < 0.01, and ***p < 0.001)

**Fig. 5:**
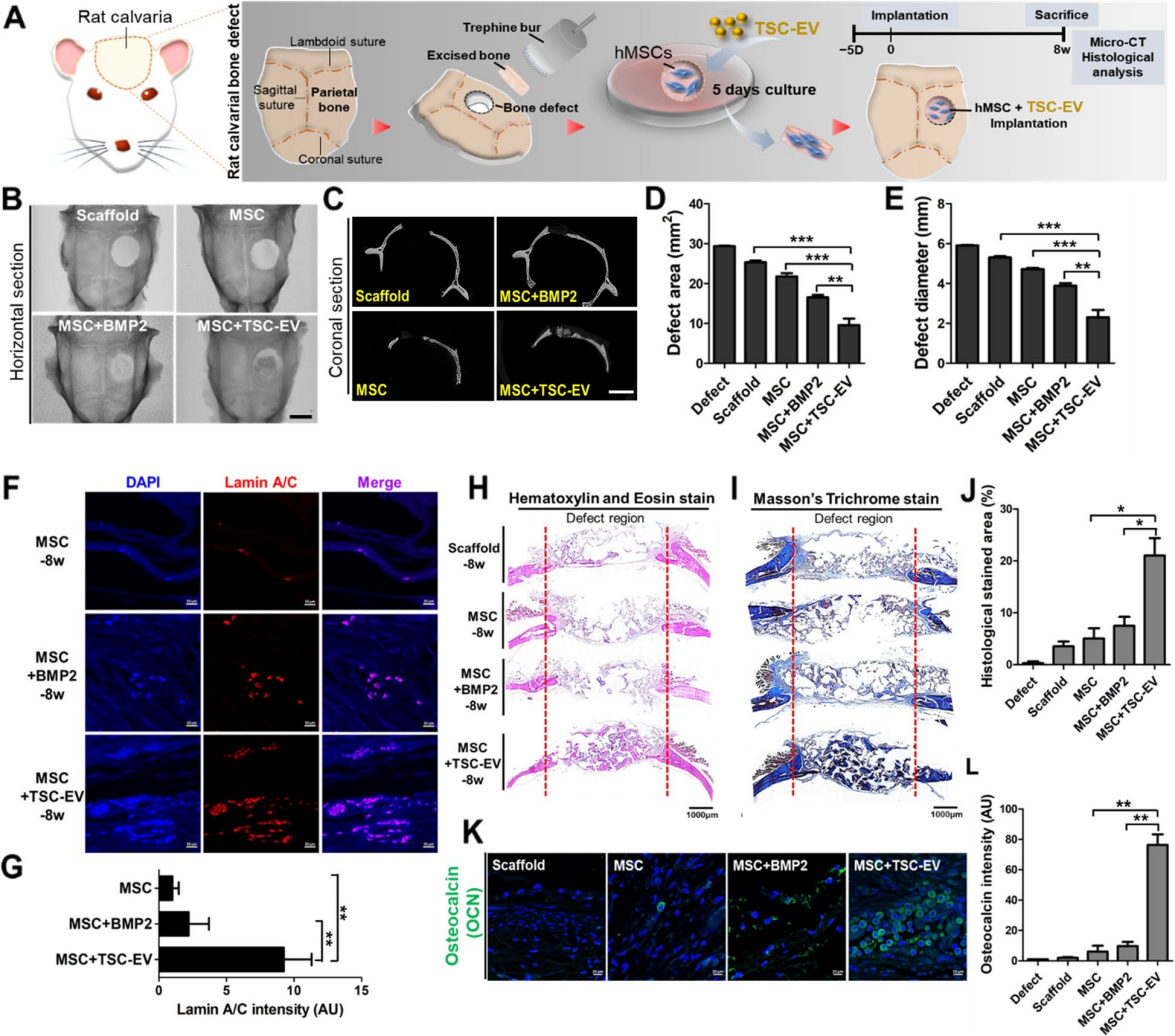
Bone regeneration effects of TSC-EVs on MSCs (**A**) Schematic representation of the experimental processes for rat calvaria bone-defect modeling with TSC-EV-treated MSC-laden scaffolds. Horizontal (**B**) and coronal (**C**) micro-CT images of calvaria bone-defect in scaffold, MSC, MSC + BMP2, and MSC + TSC-EV. The defect area (**D**) and diameter (**E**) of calvaria bone-defect were measured. Defect only of the skull bone was used as a control. **F** Representative fluorescent images of lamin A/C in MSC, MSC + BMP2, and MSC + TSC-EV laden groups. **G** The intensity of lamin A/C expression was determined by imageJ. Histological appearance of new bone formation within calvaria bone defects was analyzed by hematoxylin and eosin (**H**) and trichrome staining (**I**) in defect, scaffold, MSC, MSC + BMP2, and MSC + TSC-EV. The red line presented the boundary of the defect region. Scale bars, 1000 μm. **J** The percentage of histological stained area in defect regions determined with all groups. **K** Representative images of immunofluorescence-stained osteocalcin (green) in scaffold, MSC, MSC + BMP2, and MSC + TSC-EV. Scale bars, 10 μm. **L** Fluorescence intensity for osteocalcin was measured in each group. Data presented the mean values and SD from four to eight rat per group. (*p < 0.05, **p < 0.01, and ***p < 0.001)

To elucidate whether TSC-EVs positively regulate the osteogenesis of MSCs, MSCs were treated with TSC-EVs during in vitro osteogenesis. The results showed that TSC-EV treatment significantly increased the accumulation of ALP and calcium in MSCs in the early and late stages of osteogenesis (Fig. 4A, B). The relative expression of osteogenesis-associated genes, such as *Ibsp*, *Runx2*, *Osterix*, *Ocn*, and *Opn* were also all upregulated during osteogenesis of MSCs (Fig. 4C, D, E, F, G, H). A significant increase in mineralization was observed in TSC-EV-treated MSCs (Fig. 4I, J), and additional treatment of TSC-EVs with TSC-CM showed a synergistic effect on the ALP activity of MSCs during in vitro osteogenesis (Fig. 4K). We next evaluated the bone-forming ability of TSC-EV-treated MSCs using a rat calvarial defect model. The in vitro culture of MSCs onto scaffolds with or without TSC-EVs for 5 days took precedence in experimental processes and subsequently implanted into the rat calvarial-defect regions (Fig. 5A, S6A). Bone morphogenetic protein 2 (BMP2), an FDA-approved stimulator for bone regeneration [42], was applied to MSCs that were used as controls with scaffold-only and MSC-only groups, without TSC-EV treatment (hereafter termed the MSC + BMP2, scaffold, and MSC groups, respectively). Micro-CT evaluation was performed after 8 weeks, and regenerative bone tissues were found in the TSC-EV-treated MSCs (MSC + TSC-EV) groups compared with those in the controls (Fig. 5B, C). Quantification of defect area and diameter was significantly reduced in the MSC + TSC-EV group compared to that in the MSC + BMP2 group, which indicates an excellent regenerative effect of TSC-EVs on MSCs during bone tissue formation (Fig. 5D, E). Moreover, fluorescent signals from human-specific lamin A/C on MSCs were significantly detected in the MSC + TSC-EV group (Figure 5F, G). The results of histological analysis, including H&E and trichrome staining, also showed a significant increase in newly calcified bone tissues in the MSC + TSC-EV group compared with controls (Fig. 5H, I). Intensive staining of new bone tissue in the rat calvarial defect region in MSC + TSC-EVs clearly showed twofold higher bone accumulation than the MSC + BMP2 group (Fig. 5J). Osteocalcin (OCN), which is a bone-specific marker protein [43] was highly expressed in the MSC-laden scaffolds treated with TSC-EVs compared with the control groups (Fig. 5K, L, S6B). These results indicate that TSC-EV treatment of MSCs significantly promoted the ossification ability of MSCs, which led to rapid bone regeneration.

### TSC-EVs improve anti-senescence effects and stemness activity of MSCs

**Fig. 6:**
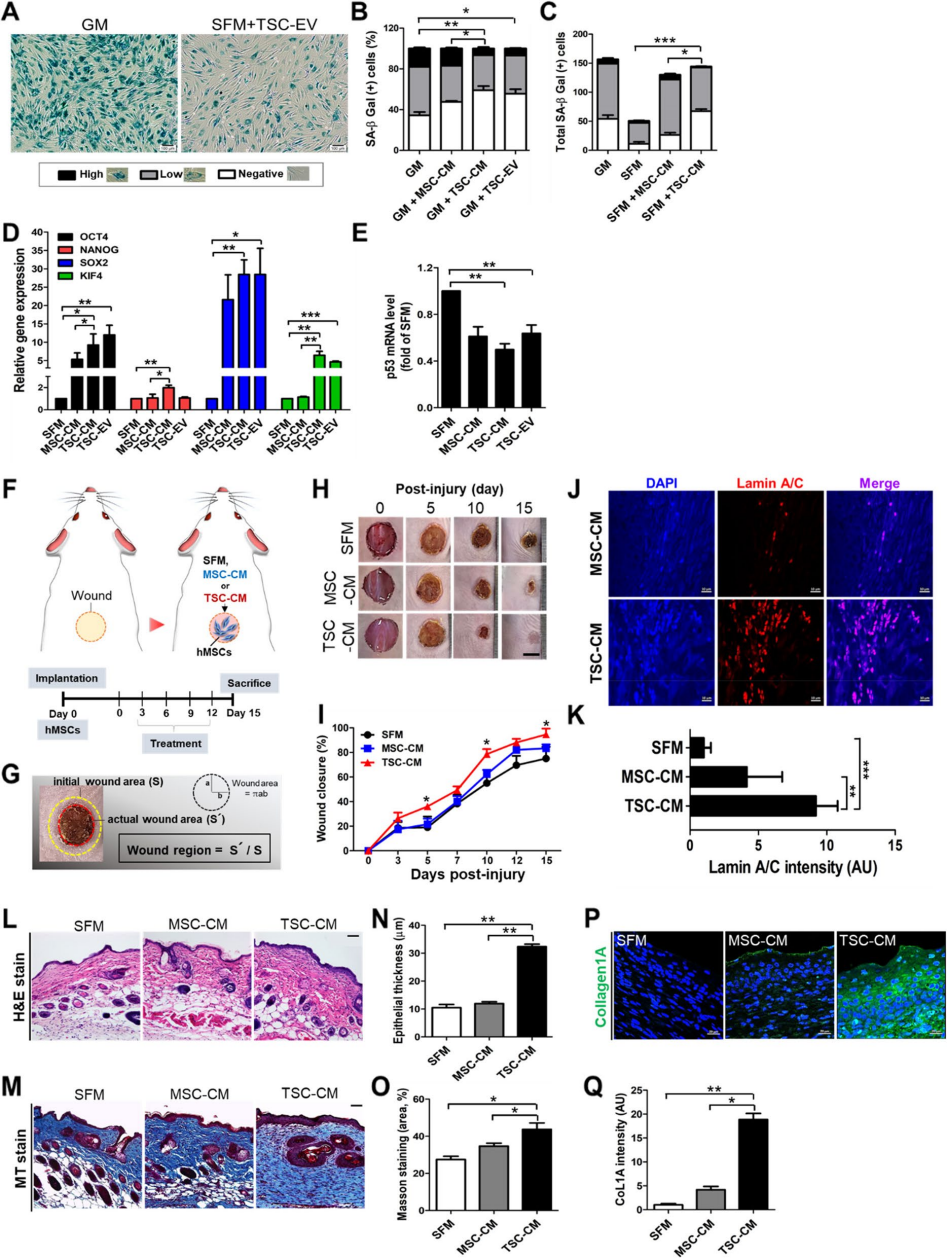
Anti-senescence and wound-healing effects of MSCs by TSC-derived secretome. The effect of TSC-CM or -EV on MSCs senescence was determined by SA-β-gal activity. **A** Representative images of SA-β-gal staining of TSC-EV-treated and untreated MSCs. Scale bars, 100 μm. SA-β -gal-positive cells in MSC-CM, TSC-CM, and -EV-treated MSCs cultured in GM **B** or SFM (**C**). **D** Relative expression levels of stemness-associated genes in MSCs treated with SFM, MSC-CM, TSC-CM, and -EV for 48 h. **E** Relative p53 mRNA levels in MSCs after 48 h incubation with SFM, MSC-CM, TSC-CM, and -EV. Error bars indicate the mean and SD. (*n* = 3; *p < 0.05, **p < 0.01, and ***p < 0.001) (**F**) Schematic illustration of experimental procedure for skin wound in mice (**G**) Relative wound region was calculated as actual wound area (red circle; S´) divided by initial wound area (yellow circle; S). **H** Representative photographs showing the wound-healing area in mice treated with SFM, MSC-CM, and TSC-CM-exposed MSCs on the skin wounds. Scale bars, 0.5 mm. **I** Quantitative analysis of wound closure percentage. **J** Representative images of human-specific lamin A/C staining with MSCs on wounded area in MSC-CM and TSC-CM treated groups. **K** The fluorescent intensity of lamin A/C was analyzed by imageJ. Histological analysis of the wound region using hematoxylin and eosin (**H**&**E**) (**L**, **N**) and Masson trichrome (MT) staining (**M**, **O**). Scale bars, 50 μm. **P**, **Q** Collagen expression (green) in the wound-healing area was determined by immunofluorescence staining. Scale bars, 20 μm. Data represent the mean ± SD of four to eight mice per group. (*p < 0.05 and **p < 0.01)

MSCs are continuously aged at multiple passages in vitro, and senescent cells have a negative effect on regenerative capacities and aberrant age-related issues, such as tumor formation [44, 45]. Next, we explored the anti-senescence effect of TSC-EVs on MSCs and their proliferation-promoting efficacy during in vitro expansion. To determine the inhibitory role of the TSC-derived secretomes on cellular senescence, late-passaged MSCs (P9-10) were treated with TSC-EVs or -CM, and SA-β-gal activity was detected. SA-β-gal staining was used as a detection method for senescent cells [46]. TSC-EVs markedly disappeared the stained area of SA-β-gal in late-passaged MSCs (Fig. 6A), and the percentage of highly stained cells was significantly reduced in the TSC-CM- and -EV-treated groups compared to those in the MSC-CM and untreated GM groups (Fig. 6B). A significant increase of SA-β-gal unstained cells was also observed in TSC-CM-treated MSCs even in SFM conditions (Fig. 6C, S7A). We further evaluated the expression levels of stemness-associated genes, including *Oct4*, *Nanog*, *Sox2*, and *Klf4*, which were all significantly elevated in TSC-CM- and -EV-treated MSCs compared to the control (Fig. 6D). Moreover, apoptosis and senescence-related p53 genes were significantly downregulated in TSC-CM- and -EV-treated MSCs (Fig. 6E). Altogether, these results demonstrated that TSC-CM and -EV treatment inhibited cellular senescence and reactivated the self-renewal activity of MSCs.

### TSC-CM enhance therapeutic potential of MSCs on skin wound

MSCs and MSC-CM are known to have regenerative potential in skin wound healing [47]. We next compared the regenerative capacity of MSC-CM and TSC-CM in an animal skin wound model. MSCs were implanted into the skin wound area with TSC-CM or MSC-CM, and then followed every 3-day treatment with each cell-derived CM for 15 days (Fig. 6F). The injured area in the SFM-only, MSC-CM, and TSC-CM-treated groups was measured (Fig. 6G), and a significant decrease in skin wounds was observed in the TSC-CM-treated group after day 10 (Fig. 6H, I, and S7B). High accumulation of MSCs at the wound site were observed in the TSC-CM-treated group using the human-specific marker lamin A/C, indicating that TSC-CM positively affected MSC viability in vivo (Fig. 6J, K). As expected, histological analysis also showed that TSC-CM treatment effectively restored injury to skin tissue, and damaged epithelial and dermal regions were remarkably healed in the TSC-CM-treated group compared to those in the controls (Fig. 6L-O and S7C, D). Additional treatment with TSC-CM stimulated the synthesis of dermal collagen, which was also detected (Fig. 6P, Q, S7E). Based on these data, we investigated the regenerative potential of MSCs resulting from the therapeutic efficacy of TSC-CM treatment during skin wound healing.

### TSC-EVs drive activation of NGF/Akt pathway in therapeutic effects of MSCs

**Fig. 7:**
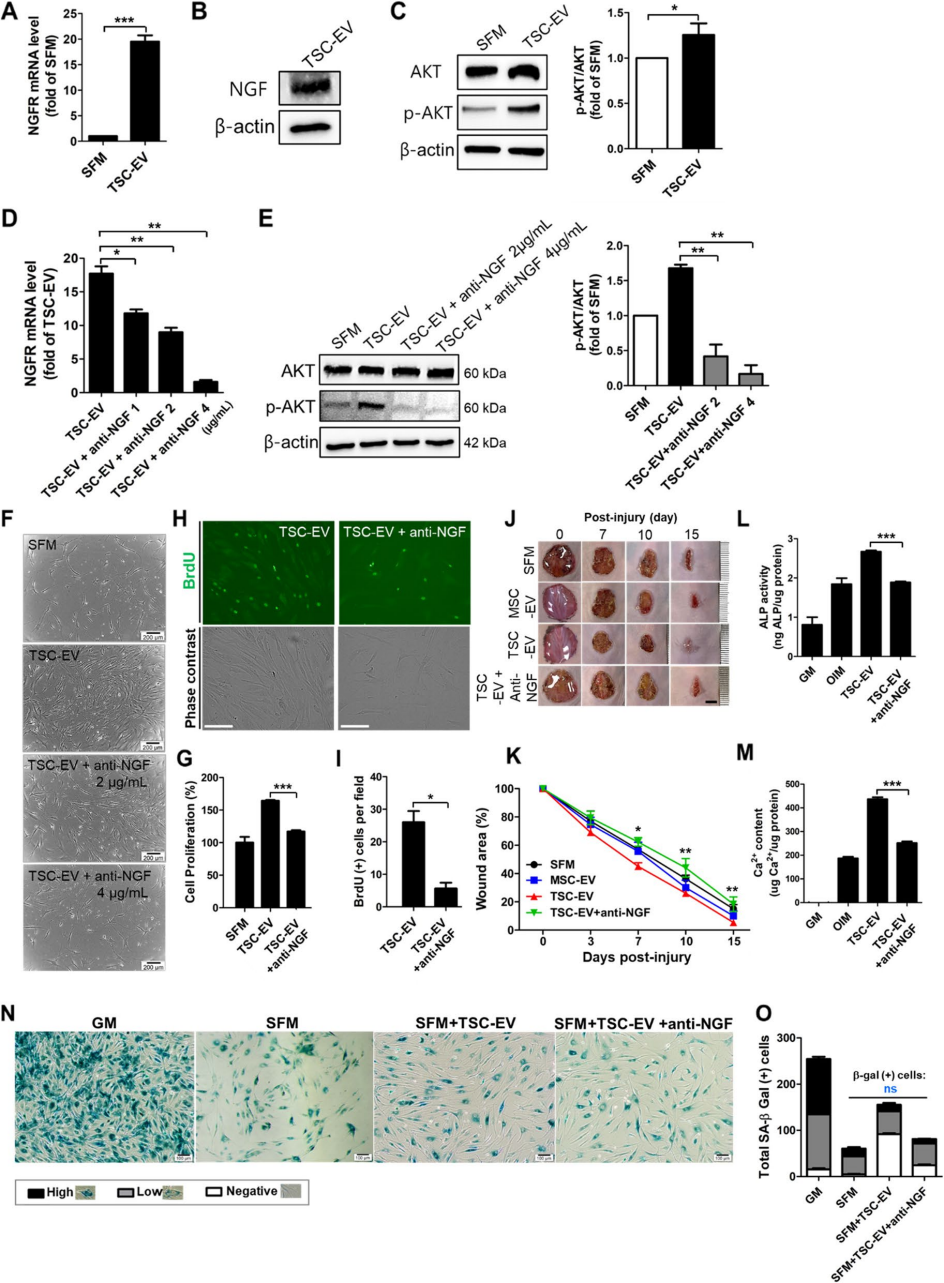
TSC-EVs induced proliferation and regenerative effects on MSCs by NGF/Akt activation. **A** NGFR mRNA level was analyzed by RT-PCR in MSCs treated with SFM or TSC-EVs for 24 h. **B** NGF in TSC-EVs was detected by western blot analysis. **C** Protein levels of Akt and p-Akt in MSCs with SFM or TSC-EVs treatment were determined by western blot and quantitated the expression level of each group. **D** Expression levels of NGFR gene and (**E**) protein levels of Akt and p-Akt were analyzed in MSCs with SFM, TSC-EV, and TSC-EV + anti-NGF. **F** Light microscopy images and (**G**) cell proliferation rate in MSCs with SFM, TSC-EV, and TSC-EV + anti-NGF. Scale bars, 200 μm. **H** Representative images of BrdU expression (green) in TSC-EV-treated MSCs with or without anti-NGF. Scale bars, 125 μm. **I** The number of BrdU-positive cells were represented by graph. **J** Representative photographs of wound scar area in mice treated with SFM, MSC-EV, TSC-EV, and TSC-EV + anti-NGF exposed MSCs. Scale bars, 0.5 mm. **K** The percentage of open area on skin wound was measured in each group. Data represented the mean and SD from four to eight mice per group. (*p < 0.05 and **p < 0.01) ALP activity (**L**) and calcium deposition (**M**) of MSCs treated with SFM, TSC-EV, and TSC-EV + anti-NGF were determined. **N** Representative images of SA-b-gal stained MSCs cultured with GM, SFM, TSC-EV, and TSC-EV + anti-NGF. Scale bars, 100 μm. **O** Stained cells were calculated in each group and there was no statistical significance in SFM, TSC-EV, and TSC-EV + anti-NGF. Error bars indicated the mean and SD. (*n* = 3; *p < 0.05, **p < 0.01, and ***p < 0.001)

Based on RNA-sequencing results in the TSC-EV-treated MSCs, we found a highly upregulated nerve growth factor receptor (NGFR) gene, which was confirmed by real-time PCR (Fig. 7A). It has been studied the effects of NGF on MSCs, NGF has the biological and therapeutic effects of MSCs, including their proliferation, anti-apoptosis, multiple-differentiation capacity [48, 49]. In particular, intracellular Akt signaling is activated by NGF-NGFR binding to MSCs, which promotes the survival, proliferation, and osteogenesis of MSCs [50–52]. Therefore, we further determined the functional role of NGF/Akt signaling in the proliferation and regenerative capacity of TSC-EVs in MSCs. As expected, NGF was detected in TSC-EVs, and the expression levels of p-Akt were increased by TSC-EV treatment of MSCs (Fig. 7B, C). Upregulated NGFR mRNA levels in TSC-EV-treated MSCs were gradually decreased by dose-dependent treatment with an antibody against NGF (anti-NGF) (Fig. 7D), and the level of p-Akt was also decreased in the TSC-EV with anti-NGF group (Fig. 7E). It is known that anti-NGF intervenes in the cellular binding of NGF-NGFR to abolish the effect of NGF on cells [53]. Pre-treatment with anti-NGF did not affect cellular toxicity in MSCs in this study (Figure S8A). These results indicated that anti-NGF neutralized NGF in TSC-EVs, which followed the suppression of NGF/Akt signaling in MSCs. Next, we verified whether NGF/Akt signaling in TSC-EV-treated MSCs is involved in the proliferation- and regeneration-promoting effects of TSC-EVs on MSCs. The percentage of cell proliferation and BrdU-positive cells were significantly reduced in the group of TSC-EVs with anti-NGF compared with only TSC-EV-treated MSCs (Fig. 7F, G, H, I). The therapeutic potential of TSC-EVs on wound healing was analyzed in our transcriptome analysis (Figs. 2 and 3), which also confirmed that anti-NGF treatment significantly inhibited the wound-healing effect of TSC-EVs (Fig. 7J, K, S8B). Furthermore, the osteogenic efficacy of TSC-EVs on MSCs also returned to the OIM group in the group of TSC-EVs with anti-NGF (Fig. 7L, M), indicating that NGF/Akt signaling offset the effect of anti-NGF treatment on TSC-EVs. However, the anti-senescence effect of TSC-EVs was not affected by NGF signal activation (Fig. 7N, O). Overall, anti-NGF treatment significantly attenuated the substantial effects of TSC-EVs on MSCs, such as growth, wound healing, and osteogenesis, suggesting that the proliferation and regenerative effects of TSC-EVs were mediated by the NGF/Akt pathway in MSCs.

## Discussion

Stem cell-derived EVs can also sufficiently modulate stem cell properties, such as stem cell maintenance, reprogramming, proliferation, differentiation, and migration abilities, and because of these effects, they could be a suitable, safe, and efficacious alternative in regenerative medicine compared to stem cell transplantation therapy [54, 55]. Specifically, EVs derived from pluripotent stem cells, such as ESCs and iPSCs, can regulate MSC properties to promote therapeutic effects [56, 57]. ESC-EVs have positive effects on the proliferation potential and senescence of MSCs [58]. Recent studies have demonstrated that EVs secreted by iPSCs can reduce aged cellular features of MSCs [59]. Both types of pluripotent stem cells can effectively rejuvenate the senescence of MSCs, but they have the potential to cause tumorigenesis. In this study, we first introduced TSC as a substantial stem cell derived from the placenta in regenerative medicine and found that TSC-EVs can significantly enhance the serum-free proliferation and regenerative capacities of MSCs.

Exosome-derived biomolecules characterize the functional capacity of parent cells, regulate intracellular communication in various cellular processes, and control cell fate [60, 61]. Particulary, microRNA-containing EVs, can modulate gene expression by regulating target mRNAs during the post-transcriptional process, thereby influencing cell fate decisions as well as overall tissue remodeling and regeneration [39, 40, 62]. Moreover, the composition and number of microRNAs in EVs vary depending on the cell type and cellular status, which can exert various biological effects [41]. Our study provides a global view of microRNA expression patterns based on TSC- and MSC-EV comparative small RNA profiling, indicating that the overall composition of microRNA cargo in TSC- and MSC-EVs was significantly different. TSC-EVs had at least 20-fold more abundant microRNAs than MSC-EVs. Furthermore, regulatory targets of four microRNAs, miR-1246, miR-1290, let-7b-5p, and miR-423-5p, are involved in specific biological processes and pathways, such as stem cell proliferation/differentiation and tissue regeneration. Conversely, the 14 most abundant microRNAs in MSC-EV targeted genes were enriched for kinase activity and cancer signaling. These results imply that TSC-EVs can exert their biological effects more effectively and rapidly during the regeneration process than MSC-EVs.

The results of the mechanistic analysis showed that the NGF/Akt pathway is involved in the regeneration effects of TSC-EV-treated MSCs. Several studies have supported that NGF-NGFR binding could be responsible for the therapeutic effects of MSCs via activation of downstream Akt signaling, which promotes the anti-apoptotic activity, proliferation, chondrogenic, and osteogenic differentiation of MSCs. [49, 52, 53, 63] In this study, NGF was detected in TSC-EVs, but not in MSC-EVs (Figure S8C). Neutralization of NGF in TSC-EVs could not induce the proliferation- and regeneration-promoting effects of MSCs. Thus, NGF is an important component that stimulates regenerative properties in MSCs as an effector cytokine in TSC-EVs. On the contrary, molecular discrepancy such as microRNA composition and insufficient NGF in MSC-EVs might not be able to provide comparative proliferation and regenerative effects compared to TSC-EVs treatment.

The correlation between NGF and adipogenesis has not been well elucidated, but DEGs in adipogenesis of MSCs, such as peroxisome proliferator-activated receptor gamma (PPARγ) and CCAAT/enhancer-binding protein (C/EBPβ), were significantly decreased in our transcriptomic analysis. In fact, TSC-CM inhibited adipogenic differentiation of MSCs in our previous study [64]. We assumed that components including NGF and microRNAs in TSC-EVs might commit the cell fate of MSCs to osteogenesis via upregulation of the osteogenic-related RUNX2 transcription factor, TGFβ/BMP, and Wnt signaling, which simultaneously followed the suppression of adipogenesis of MSCs [65, 66].

TSC-EVs were found to be an effective regeneration-inducing biomaterial in this study. Herein, two technical approaches of this study can be suggested. First, TSC-EVs can be used in regenerative medicine in much smaller amounts than MSC-EVs because they contain concentrated functional microRNAs for tissue regeneration. The high proportion of these four microRNAs in TSC-EVs could further use in the field of regenerative medicine to achieve specific outcomes in treated patients. Recently, Yue et al. introduced a dual microRNA-triggered combination therapy for cancer in which two microRNAs (miR-21 and miR-10b) were assembled into doxorubicin nanocarriers to improve the effects of chemotherapy [67]. Thus, our findings present a potential strategy based on using specific combinations of four microRNAs in TSC-EVs with regenerative nanoparticles in future studies. Secondly, TSC-EVs offers excellent efficacy for new bone formation and cutaneous healing of the skin after MSC implantation. The direct transplantation of MSCs and EVs is still the primary method for MSC therapy in the clinic. However, it has limitations such as the rapid release of EVs, poor survival rate of transplanted cells, and unintended localization of cells adjacent to the wound area. Kim et al. showed that MSC-EVs-coated 3D scaffolds not only induced the proliferation of MSCs but also subsequently enhanced the efficacy of bone healing [68]. Both stem cells and EVs simultaneously carried bioengineered scaffolds that effectively transplanted surviving cells with EV bioactive stimulator to injury sites, which could accelerate bone regeneration. Regarding the successful delivery of TSC-EVs to implant areas, the development of TSC-EV-coated scaffolds can be an excellent approach to enhance the effectiveness of MSC-based tissue regeneration. Moreover, sufficient MSCs could be readily obtained in the TSC-EV-coated scaffold with a xeno-free in vitro culture system and then sufficient cells could be transferred to the graft area via the scaffold without loss of MSCs. Thus, the TSC-EV-coated bioengineer scaffold could offer a simple and effective application in the clinical use of exosomes for tissue regeneration.

A limitation of this study was that human TSCs entail ethical issues because they are derived from early embryos. Moreover, the stemness of TSCs is difficult to maintain in vitro because of their rapid differentiation properties. Therefore, analyzing EV components derived from TSCs may help screen for essential regulatory factors to promote the regeneration efficacy of MSCs. Future studies should explore the regeneration factors in TSC-EVs using multi-omics analysis and EV databases to develop the application of EV-based regenerative therapy.

## Conclusion

In summary, delivering TSC-EVs into MSCs boosts the regenerative properties of MSCs via NGF/Akt signaling, consequently promoting therapeutic efficacy in bone defects and skin injury. Additional studies on the TSC-EV-mediated therapeutic effects in various tissue diseases, including nerve defects and rheumatoid arthritis, are needed to expand the application of TSC-EVs in regenerative medicine. Our results provide novel insights regarding the use of EVs derived from TSCs in MSC-based therapy.

## Supplementary Information

**Additional file 1:** Supplemental Methods. **Supplementary Figure 1. **Flow chart of transcriptomic analysis in this study. **Supplementary Figure 2. **Characterization of TSC-EV and MSC-EV. **Supplementary Figure 3. **Increased proliferation rate of human MSCs via TSC-derived CM and EV. **Supplementary Figure 4. **Significant biological processes and pathway of four microRNAs primed by TSC-EVs. **Supplementary Figure 5. **DEGs in significant KEGG pathways and enriched GO terms in TSC-EV-treated MSCs. **Supplementary Figure 6. **Bone regeneration effect of TSC-EVs. **Supplementary Figure 7. **Anti-senescence and wound-healing effects of TSC-derived secretomes. **Supplementary Figure 8. **Anti-NGF effect on MSCs and TSC-EV-treated MSCs. **Supplementary Table 1. **Primer sequences used in qRT-PCR. **Supplementary Table 2. **List of antibodies and chemical materials used in this study. **Supplementary Table 3. **Statistics results of microRNA analysis data. **Supplementary Excel Table 1. **microRNA total read counts in TSC- and MSC-EV. Source Data of Western Blotting.

## Data Availability

The data of this study are available from the corresponding author upon reasonable request.

## Abbrevitions

AD-MSCs: Adipose tissue-derived mesenchymal stem cells
ATCC: American type culture collection
ALP: Alkaline phosphatase
BrdU: Bromodeoxyuridine
BM-MSCs: Bone marrow-derived mesenchymal stem cells
BMP2: Bone morphogenetic protein 2
CCK8: Cell counting Kit 8
C/EBPβ: CCAAT/enhancer-binding protein
CM: Conditioned medium
DAVID: Database for annotation, visualization, and integrated discovery
DEGs: Differentially expressed genes
ESCs: Embryonic stem cells
EV: Extracellular vesicles
FBS: Fetal bovine serum
FDR: False discovery rate
GO: Gene ontology
GM: Growth medium
GSEA: Gene set enrichment analysis
H&E: Hematoxylin and eosin
IACUC: Institutional animal care and use committee
iPSCs: Induced pluripotent stem cells
KEGG: Kyoto encyclopedia of genes and genomes
MDS: Multidimensional scaling
micro-CT: Microcomputed tomography
MSCs: Mesenchymal stem cells
MT: Masson’s trichrome
NGF: Nerve growth factor
NGFR: Nerve growth factor receptor
NTA: Nanoparticle tracking analysis
OCN: Osteocalcin
OPN: Osteopontin
OSX: Osterix
OIM: Osteogenic induction medium
PPAR-γ: Peroxisome proliferator-activated receptor-gamma
RNA-seq: RNA-sequencing
rRNA: Ribosomal RNA
SFM: Serum-free medium
snRNA: Small nuclear R
TEM: Transmission electron microscopy
TSCs: Trophoblast stem cells
TSC-EVs: Trophoblast stem cells-derived extracellular vesicles
UC-MSCs: Umbilical cord-derived mesenchymal stem cells

## References

[1] Kolios G, Moodley Y (2013). Introduction to stem cells and regenerative medicine. Respiration.

[2] Rajabzadeh N, Fathi E, Farahzadi R (2019). Stem cell-based regenerative medicine. Stem Cell Investig.

[3] Mahla RS (2016). Stem cells applications in regenerative medicine and disease therapeutics. Int J Cell Biol.

[4] Musiał-Wysocka A, Kot M, Majka M (2019). The pros and cons of mesenchymal stem cell-based therapies. Cell Transplant.

[5] Zhou T (2021). Challenges and advances in clinical applications of mesenchymal stromal cells. J Hematol O.

[6] Ocansey DKQ (2020). Improved therapeutics of modified mesenchymal stem cells: an update. J Transl Med.

[7] Wei W, Huang Y, Li D, Gou HF, Wang W (2018). Improved therapeutic potential of MSCs by genetic modification. Gene Ther.

[8] Hu C, Li L (2018). Preconditioning influences mesenchymal stem cell properties in vitro and in vivo. J Cell Mol Med.

[9] Li Q, Zhang A, Tao C, Li X, Jin P (2013). The role of SDF-1-CXCR4/CXCR7 axis in biological behaviors of adipose tissue-derived mesenchymal stem cells in vitro. Biochem Biophys Res Commun.

[10] Yum S (2017). Minoxidil induction of VEGF is mediated by inhibition of HIF-prolyl hydroxylase. Int J Mol Sci.

[11] Kim DS (2016). Effect of low oxygen tension on the biological characteristics of human bone marrow mesenchymal stem cells. Cell Stress Chaperones.

[12] Okae H (2018). Derivation of human trophoblast stem cells. Cell Stem Cell.

[13] Castel G (2020). Induction of human trophoblast stem cells from somatic cells and pluripotent stem cells. Cell Rep.

[14] Valzacchi GMR, Odetto D, Chacon CB, Wernicke A, Xiang Y (2020). Placental site trophoblastic disease. Int J Gynecol Cancer.

[15] Red-Horse K (2004). Trophoblast differentiation during embryo implantation and formation of the maternal-fetal interface. J Clin Invest.

[16] Salomon C (2014). Extravillous trophoblast cells-derived exosomes promote vascular smooth muscle cell migration. Front Pharmacol.

[17] Su Y (2022). Exosomes derived from placental trophoblast cells regulate endometrial epithelial receptivity in dairy cows during pregnancy. J Reprod Dev.

[18] Shi S (2021). Placental trophoblast cell-derived exosomal microRNA-1290 promotes the interaction between endometrium and embryo by targeting LHX6. Mol Ther Nucleic Acids.

[19] Hoshino A (2015). Tumour exosome integrins determine organotropic metastasis. Nature.

[20] Kozomara A, Griffiths-Jones S (2014). miRBase: annotating high confidence microRNAs using deep sequencing data. Nucleic Acids Res.

[21] Petrov AI (2017). RNAcentral: a comprehensive database of non-coding RNA sequences. Nucleic Acids Res.

[22] Dobin A (2012). STAR: ultrafast universal RNA-seq aligner. Bioinformatics.

[23] Friedländer MR, Mackowiak SD, Li N, Chen W, Rajewsky N (2012). miRDeep2 accurately identifies known and hundreds of novel microRNA genes in seven animal clades. Nucleic Acids Res.

[24] Langmead B, Salzberg SL (2012). Fast gapped-read alignment with Bowtie 2. Nat Methods.

[25] Tokar T (2018). mirDIP 4.1-integrative database of human microRNA target predictions. Nucleic Acids Res.

[26] Martin JA, Wang Z (2011). Next-generation transcriptome assembly. Nat Rev Genet.

[27] Kim D, Langmead B, Salzberg SL (2015). HISAT: a fast spliced aligner with low memory requirements. Nat Methods.

[28] Kovaka S (2019). Transcriptome assembly from long-read RNA-seq alignments with StringTie2. Genome Biol.

[29] da Huang W, Sherman BT, Lempicki RA (2009). Systematic and integrative analysis of large gene lists using DAVID bioinformatics resources. Nat Protoc.

[30] Kanehisa M, Goto S (2000). KEGG: kyoto encyclopedia of genes and genomes. Nucleic Acids Res.

[31] Subramanian A (2005). Gene set enrichment analysis: a knowledge-based approach for interpreting genome-wide expression profiles. Proc Natl Acad Sci U S A.

[32] Mi H, Thomas P (2009). PANTHER pathway: an ontology-based pathway database coupled with data analysis tools. Methods Mol Biol.

[33] Bindea G (2009). ClueGO: a Cytoscape plug-in to decipher functionally grouped gene ontology and pathway annotation networks. Bioinformatics.

[34] Szklarczyk D (2019). STRING v11: protein-protein association networks with increased coverage, supporting functional discovery in genome-wide experimental datasets. Nucleic Acids Res.

[35] Shannon P (2003). Cytoscape: a software environment for integrated models of biomolecular interaction networks. Genome Res.

[36] Wang X, Ge J, Tredget EE, Wu Y (2013). The mouse excisional wound splinting model, including applications for stem cell transplantation. Nat Protoc.

[37] Premarathna AD (2021). In vitro and in vivo evaluation of the wound healing properties and safety assessment of two seaweeds (Sargassum ilicifolium and Ulva lactuca). Biochem Biophys Rep.

[38] Livak KJ, Schmittgen TD (2001). Analysis of relative gene expression data using real-time quantitative PCR and the 2(-Delta Delta C(T)) Method. Methods.

[39] Foo JB (2021). Mesenchymal stem cell-derived exosomes and MicroRNAs in cartilage regeneration: biogenesis, efficacy, miRNA enrichment and delivery. Pharmaceuticals (Basel).

[40] Nasser MI (2021). Mesenchymal stem cell-derived exosome microRNA as therapy for cardiac ischemic injury. Biomed Pharmacother.

[41] Teruel-Montoya R (2014). MicroRNA expression differences in human hematopoietic cell lineages enable regulated transgene expression. PLoS One.

[42] Garrison KR (2010). Bone morphogenetic protein (BMP) for fracture healing in adults. Cochrane Database Syst Rev.

[43] Delmas PD, Demiaux B, Malaval L, Chapuy MC (1986). Meunier PJ [Osteocalcin (or bone gla-protein), a new biological marker for studying bone pathology]. Presse Med.

[44] Yang YK (2018). Aging of mesenchymal stem cells: Implication in regenerative medicine. Regen Ther.

[45] Mojsilović S (2021). Tumorigenic aspects of MSC senescence-implication in cancer development and therapy. J Pers Med.

[46] Lee BY (2006). Senescence-associated beta-galactosidase is lysosomal beta-galactosidase. Aging Cell.

[47] Guillamat-Prats R (2021). The role of MSC in wound healing, scarring and regeneration. Cells.

[48] Li Y, Wang S, Xiao Y, Liu B, Pang J (2021). Nerve growth factor enhances the therapeutic effect of mesenchymal stem cells on diabetic periodontitis. Exp Ther Med.

[49] Zha K (2021). Nerve growth factor (NGF) and NGF receptors in mesenchymal stem/stromal cells: impact on potential therapies. Stem Cells Transl Med.

[50] Gharibi B, Ghuman MS, Hughes FJ (2012). Akt- and Erk-mediated regulation of proliferation and differentiation during PDGFRβ-induced MSC self-renewal. J Cell Mol Med.

[51] Wang Q (2019). NGF protects bone marrow mesenchymal stem cells against 2,5-hexanedione-induced apoptosis in vitro via Akt/Bad signal pathway. Mol Cell Biochem.

[52] Cui GS, Zeng JY, Zhang J, Lu R (2018). Effect of nerve growth factor on osteogenic potential of type 2 diabetic mice bone marrow stromal cell in vitro. Zhonghua Kou Qiang Yi Xue Za Zhi.

[53] Bai Q (2020). NGF mediates protection of mesenchymal stem cells-conditioned medium against 2,5-hexanedione-induced apoptosis of VSC4.1 cells via Akt/Bad pathway. Mol Cell Biochem..

[54] Riazifar M, Pone EJ, Lötvall J, Zhao W (2017). Stem Cell Extracellular Vesicles: Extended Messages of Regeneration. Annu Rev Pharmacol Toxicol.

[55] Nikfarjam S, Rezaie J, Zolbanin NM, Jafari R (2020). Mesenchymal stem cell derived-exosomes: a modern approach in translational medicine. J Transl Med.

[56] Xia Y (2020). Small extracellular vesicles secreted by human iPSC-derived MSC enhance angiogenesis through inhibiting STAT3-dependent autophagy in ischemic stroke. Stem Cell Res.

[57] Khan M (2015). Embryonic stem cell-derived exosomes promote endogenous repair mechanisms and enhance cardiac function following myocardial infarction. Circ Res.

[58] Zhang Y (2019). Embryonic stem cell-derived extracellular vesicles enhance the therapeutic effect of mesenchymal stem cells. Theranostics.

[59] Liu S (2019). Highly purified human extracellular vesicles produced by stem cells alleviate aging cellular phenotypes of senescent human cells. Stem Cells.

[60] Zhang Y, Liu Y, Liu H, Tang WH (2019). Exosomes: biogenesis, biologic function and clinical potential. Cell Biosci.

[61] Hamzah RN, Alghazali KM, Biris AS, Griffin RJ (2021). Exosome traceability and cell source dependence on composition and cell-cell cross talk. Int J Mol Sci.

[62] Shirazi S (2021). The importance of cellular and exosomal miRNAs in mesenchymal stem cell osteoblastic differentiation. Sci Rep.

[63] Lee HH (2013). Hypoxia enhances chondrogenesis and prevents terminal differentiation through PI3K/Akt/FoxO dependent anti-apoptotic effect. Sci Rep.

[64] Go YY, Lee CM, Chae SW, Song JJ (2022). Osteogenic efficacy of human trophoblasts-derived conditioned medium on mesenchymal stem cells. Int J Mol Sci.

[65] Chen Q (2016). Fate decision of mesenchymal stem cells: adipocytes or osteoblasts?. Cell Death Differ.

[66] Hoshiba T, Kawazoe N, Chen G (2012). The balance of osteogenic and adipogenic differentiation in human mesenchymal stem cells by matrices that mimic stepwise tissue development. Biomaterials.

[67] Yue R, Chen M, Ma N (2020). Dual MicroRNA-Triggered Drug Release System for Combined Chemotherapy and Gene Therapy with Logic Operation. ACS Appl Mater Interfaces.

[68] Kim DK, Lee S, Kim M, Jeong Y, Lee S (2021). Exosome-coated silk fibroin 3D-scaffold for inducing osteogenic differentiation of bone marrow derived mesenchymal stem cells. Chem Eng J.

