# Supplementary material for: Regenerative capacity of trophoblast stem cell-derived extracellular vesicles on mesenchymal stem cells
Source: Biomater Res. 2023 Jun 27;27:62. doi: 10.1186/s40824-023-00396-5 (PMC10304624; doi:10.1186/s40824-023-00396-5)
Supplement: Supplementary file 1 — Additional file 1: Supplemental Methods. Supplementary Figure 1. Flow chart of transcriptomic analysis in this study. Supplementary Figure 2. Characterization of TSC-EV and MSC-EV. Supplementary Figure 3. Increased proliferation rate of human MSCs via TSC-derived CM and EV. Supplementary Figure 4. Significant biological processes and pathway of four microRNAs primed by TSC-EVs. Supplementary Figure 5. DEGs in significant KEGG pathways and enriched GO terms in TSC-EV-treated MSCs. Supplementary Figure 6. Bone regeneration effect of TSC-EVs. Supplementary Figure 7. Anti-senescence and wound-healing effects of TSC-derived secretomes. Supplementary Figure 8. Anti-NGF effect on MSCs and TSC-EV-treated MSCs. Supplementary Table 1. Primer sequences used in qRT-PCR. Supplementary Table 2. List of antibodies and chemical materials used in this study. Supplementary Table 3. Statistics results of microRNA analysis data. Supplementary Excel Table 1. microRNA total read counts in TSC- and MSC-EV. Source Data of Western Blotting. [file 40824_2023_396_MOESM1_ESM.zip › Supplemental Methods_ESM.pdf]

Supplementary Information

Regenerative capacity of trophoblast stem cell-derived extracellular vesicles on mesenchymal stem cells

Yoon Young Go, Chan Mi Lee, Sung-won Chae, Jae-Jun Song*

Supplementary Methods

Nanoparticle tracking analysis

Nanoparticle tracking analysis (NTA) was used to detect the size and concentration of isolated exosomes using a NanoSight™ LM10-HS10 system (NanoSight Amesbury, UK). Each EV sample was diluted with PBS in ration of 1:100 to achieve a measurable concentration, and three recordings were made in 30 s. Based on captured images, the average size and concentration of the EVs were determined using a monochromatic laser beam (405 nm) and NanoSight™ tracking software (version 3.0).

Transmission electron microscopy

Ten microliters of trophoblast stem cell-derived extracellular vesicles (TSC-EVs) and mesenchymal stem cell (MSC)-EVs (10^6 particles/ml) was loaded onto a carbon film grid and then negatively stained with uranyl acetate solution for 1 min. The stained EVs were observed under energy-filter TEM (LIBRA 120, Carl Zeiss, Germany).
