# Supplementary material for: Regenerative capacity of trophoblast stem cell-derived extracellular vesicles on mesenchymal stem cells
Source: Biomater Res. 2023 Jun 27;27:62. doi: 10.1186/s40824-023-00396-5 (PMC10304624; doi:10.1186/s40824-023-00396-5)

Supplementary Figure 1

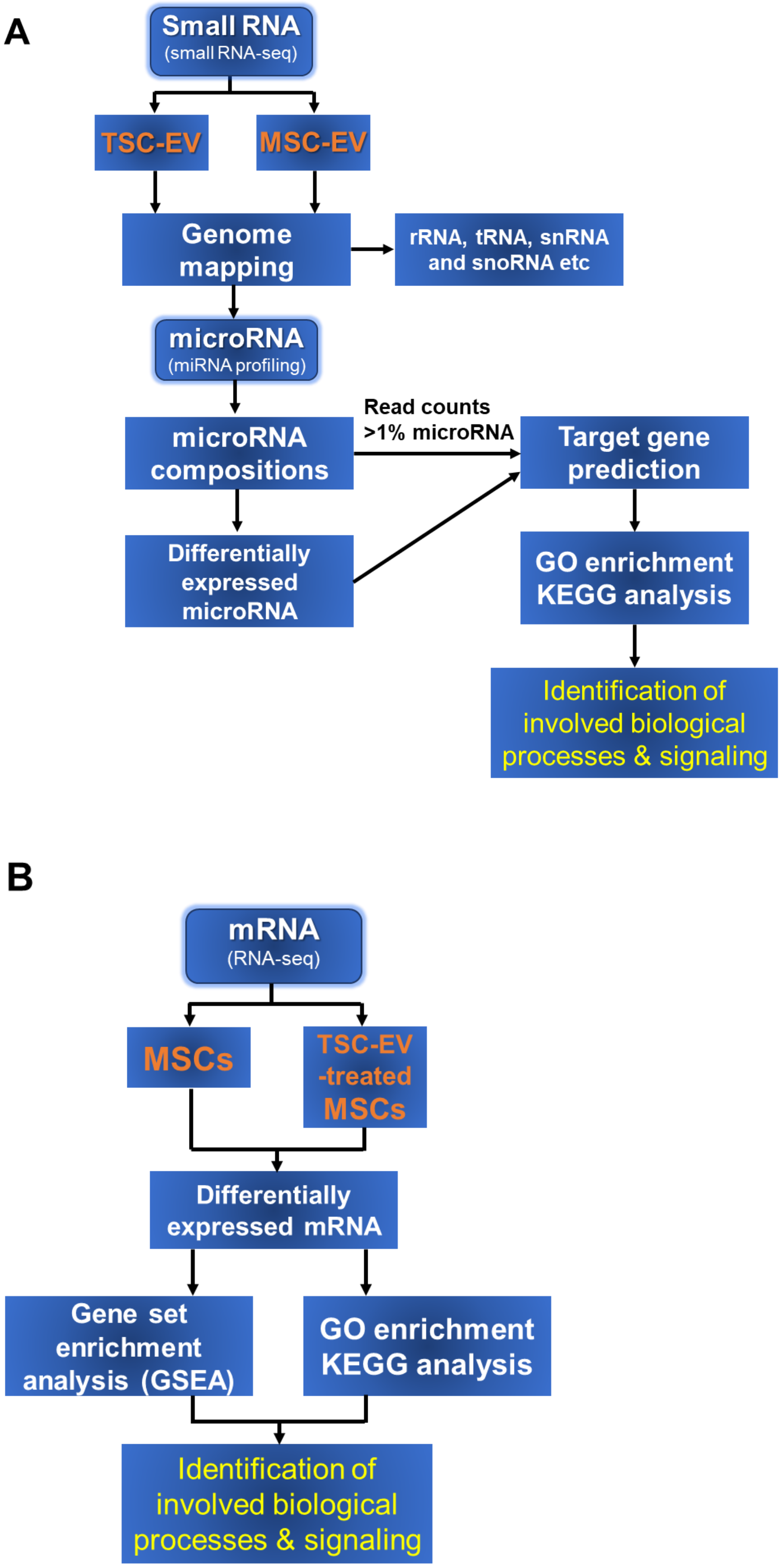

Supplementary Figure 1. Flow chart of transcriptomic analysis in this study. (A) small RNA sequencing including microRNA analysis between TSCs and MSCs. (B) mRNA sequencing of MSCs and TSC-EV-treated MSCs. Biological replicate, n=3.

Supplementary Figure 2

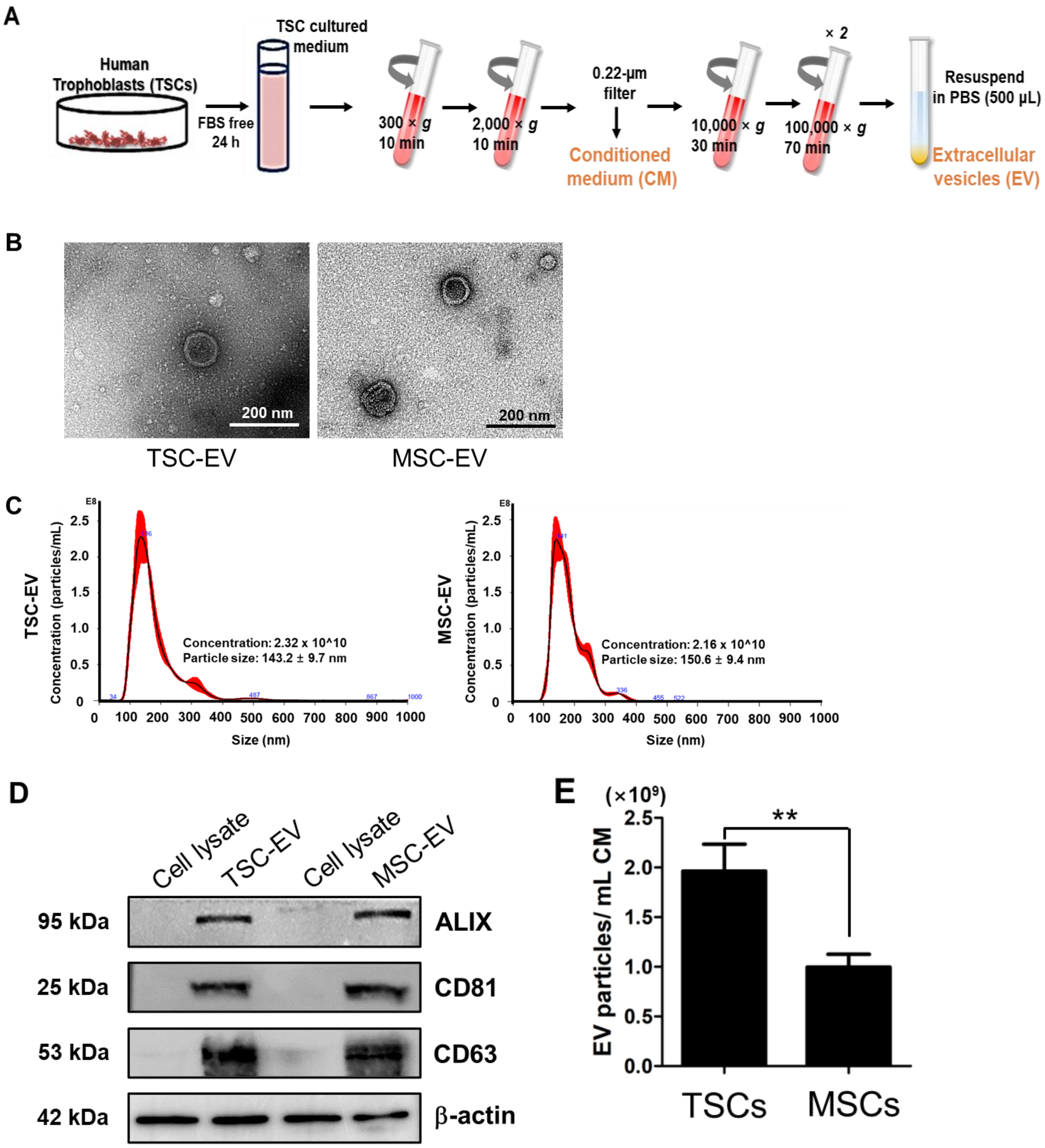

Supplementary Figure 2. Characterization of TSC-EV and MSC-EV. (A) Schematic illustration of isolated EVs from CM of cells cultures by ultracentrifugation. (B) Representative images of TSC-EV or MSC-EV using TME. Scale bars, 200 nm. (C) NTA data show the concentration and size of EVs derived from each stem cells cultures; (n=3). (D) The protein levels of ALIX, CD 81 and CD 63 in EVs and cell lysates are determined using western blotting; (n=3). (E) The yield of EVs from TSCs and MSCs are presented as results of NTA (EV particles/ 1mL of CM).

Supplementary Figure 3

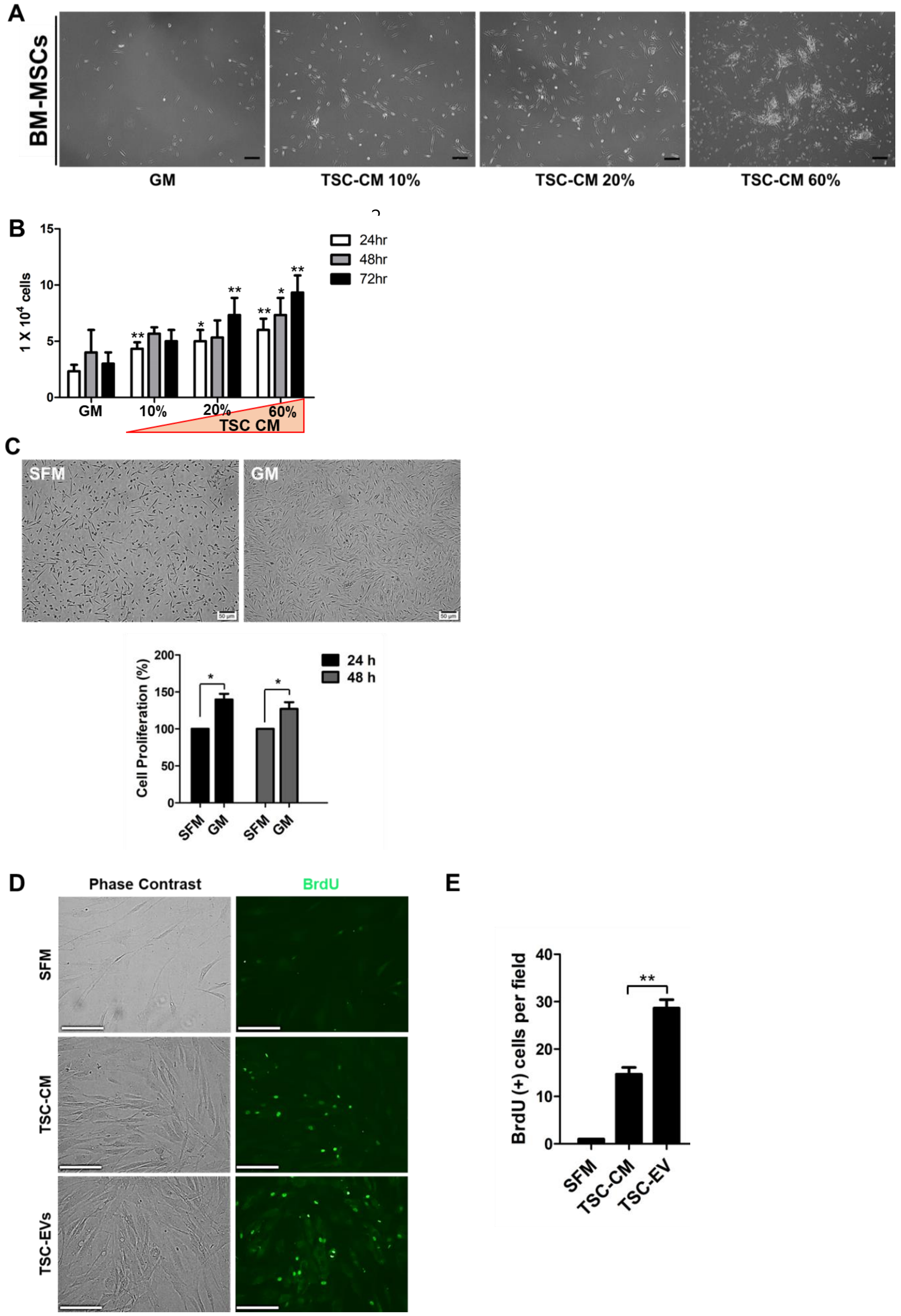

Supplementary Figure 3. Increased proliferation rate of human MSCs via TSC-derived CM and EV. A light Microscopy images (A) and cell counting analysis (B) show the increase of MSCs by TSC-CM treatment. Scale bars, 50 μm. (C) The morphology of MSCs cultured in SFM or GM. Scale bars, 50 μm. The proliferation rate of MSCs cultured in SFM and GM was determined by CCK8 at 24 and 48 h. (D) BM-MSCs were cultured with TSC-CM or TSC-EVs for 24 h in SFM and determined BrdU expression (green). The intensity of BrdU fluorescence was analyzed. MSCs culture with SFM-only was used as control. Data present the mean and SD. (n=3; **p*<0.05, ***p*<0.01, and ****p*<0.001)

Supplementary Figure 4

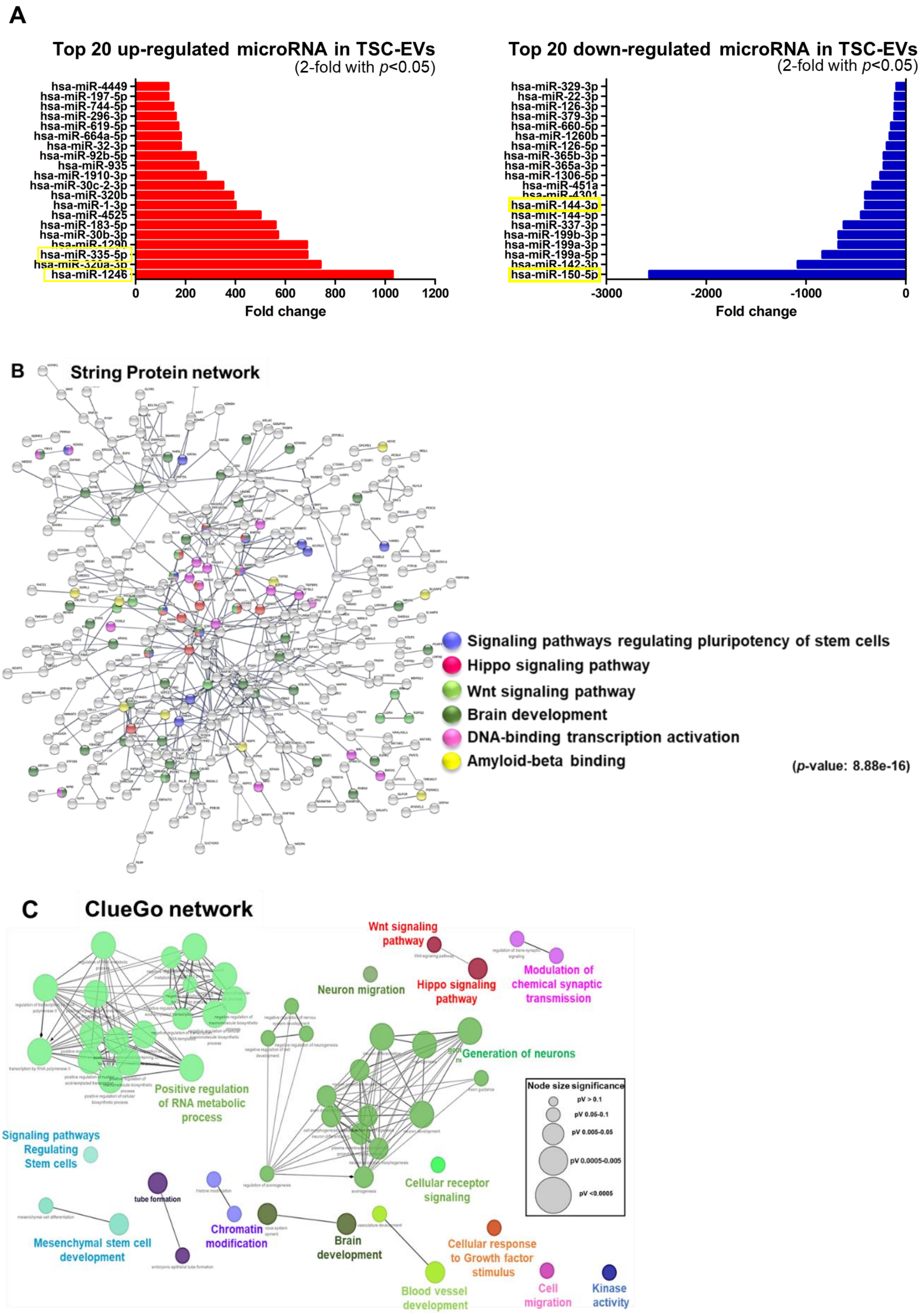

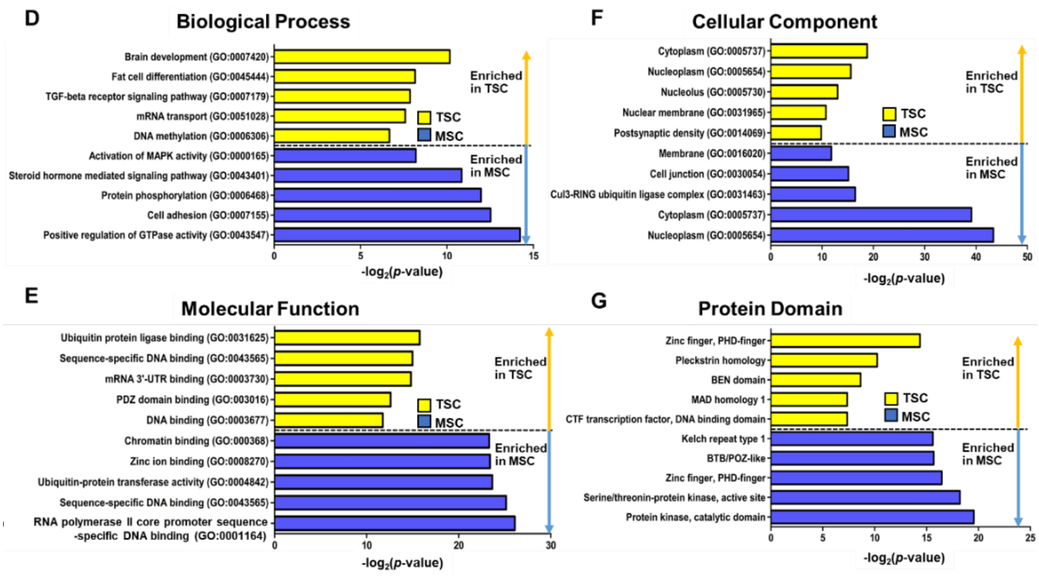

Supplementary Figure 4. Significant biological processes and pathway of four microRNAs primed by TSC-EVs. (A) The top 20 up- and down-regulated microRNAs in TSC-EVs compared to those of MSC-EVs. The yellow box indicated the randomly selected four microRNAs for quantitative RT-PCR. (B) STRING analysis presented a global network view based on target protein-PPI with six functional clusters regulated by predicted targets of four overexpressed microRNAs in TSC-EV. Targets associated with each cluster are presented in a different color. Significantly high confidence (0.7) was adjusted, and the PPI enrichment p-value was found to be 8.88e-16. (C) Functional annotations of target molecules from four microRNAs overexpressed in TSC-EV are analyzed using ClueGO (p<0.05). Node size indicates the statistical significance. DAVID analysis revealed a significant change in enriched GO terms, including biological process (D), molecular function (E), cellular component (F), and protein domain (G), by predicted targets of the most abundant microRNAs of TSC- and mesenchymal stem cell (MSC)-EV (yellow, enriched in TSC-EV; blue, enriched in MSC-EV).

Supplementary Figure 5

A Neuroactive ligand-receptor interaction

Gene symbol	Description	FC
NPY4R	neuropeptide Y receptor Y4	7.019004
NPY4R2	neuropeptide Y receptor Y4-2	5.052963
CCKAR	cholecystokinin A receptor	3.750538
GAL	galanin and GMAP prepropeptide	2.967174
GRM1	glutamate metabotropic receptor 1	2.819476
PTGIR	prostaglandin I2 receptor	2.358799
FPR1	formyl peptide receptor 1	2.066465
P2RY1	purinergic receptor P2Y1	-2.35967
S1PR3	sphingosine-1-phosphate receptor 3	-2.36627
RXFP1	relaxin family peptide receptor 1	-2.41768
GRIN2A	glutamate ionotropic receptor NMDA	-2.67432
BDKRB2	bradykinin receptor B2	-2.75553
APLN	apelin	-2.7916

B Cytokine receptor interaction

Gene symbol	Description	FC
NGFR	nerve growth factor receptor	11.65342
IL6	interleukin 6	9.287773
COL4A3	collagen type IV alpha 3 chain	2.43906
IL7R	interleukin 7 receptor	2.398263
COL4A4	collagen type IV alpha 4 chain	2.263106
ANGPT4	angiopoietin 4	2.159209
ANGPT2	angiopoietin 2	-2.04938
IL6R	interleukin 6 receptor	-2.08107
LAMA3	laminin subunit alpha 3	-2.16661
TNXB	tenascin XB	-2.64076

C PI3K-Akt signaling

Gene symbol	Description	FC
NGFR	nerve growth factor receptor	11.65342
IL6	interleukin 6	9.287773
INHBE	inhibin subunit beta E	3.1402
CCL2	C-C motif chemokine ligand 2	2.830082
IL11	interleukin 11	2.760181
LIF	LIF interleukin 6 family cytokine	2.735027
IL7R	interleukin 7 receptor	2.398263
IL21R	interleukin 21 receptor	2.095332
IL6R	interleukin 6 receptor	-2.08107
IL17RB	interleukin 17 receptor B	-2.33484
IL22RA1	interleukin 22 receptor subunit alpha	-2.44912

D

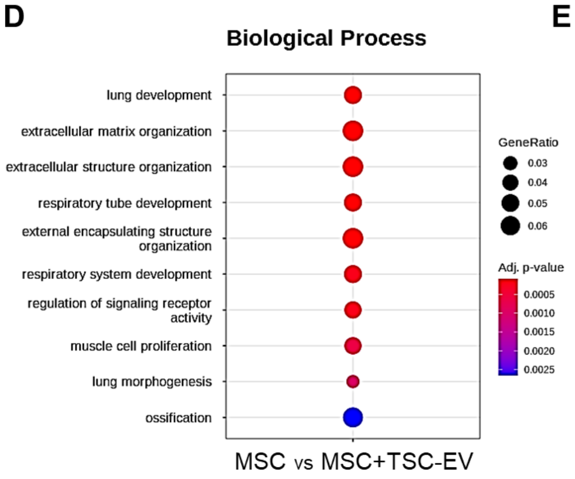

E

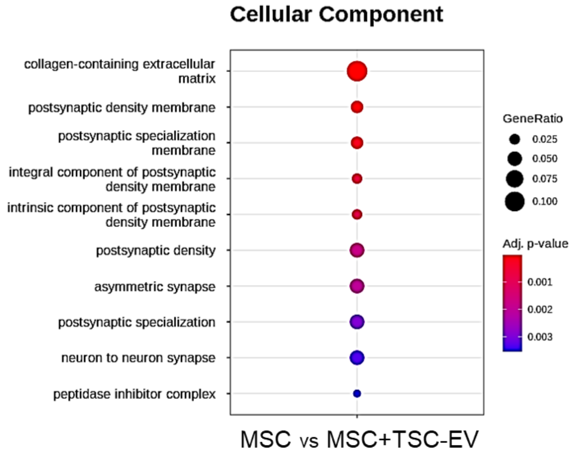

Supplementary Figure 5. DEGs in significant KEGG pathways and enriched GO terms in TSC-EV-treated MSCs. (A-C) Differentially up- and downregulated genes in significant biological processes or pathways in gene expression profile of TSC-EV-

treated MSCs group. The list of DEGs is represented by FC value. The enriched biological process (D) and cellular component (E) in TSC-EV-treated MSCs is showed with adjusted p-value and gene expression ratio.

Supplementary Figure 6

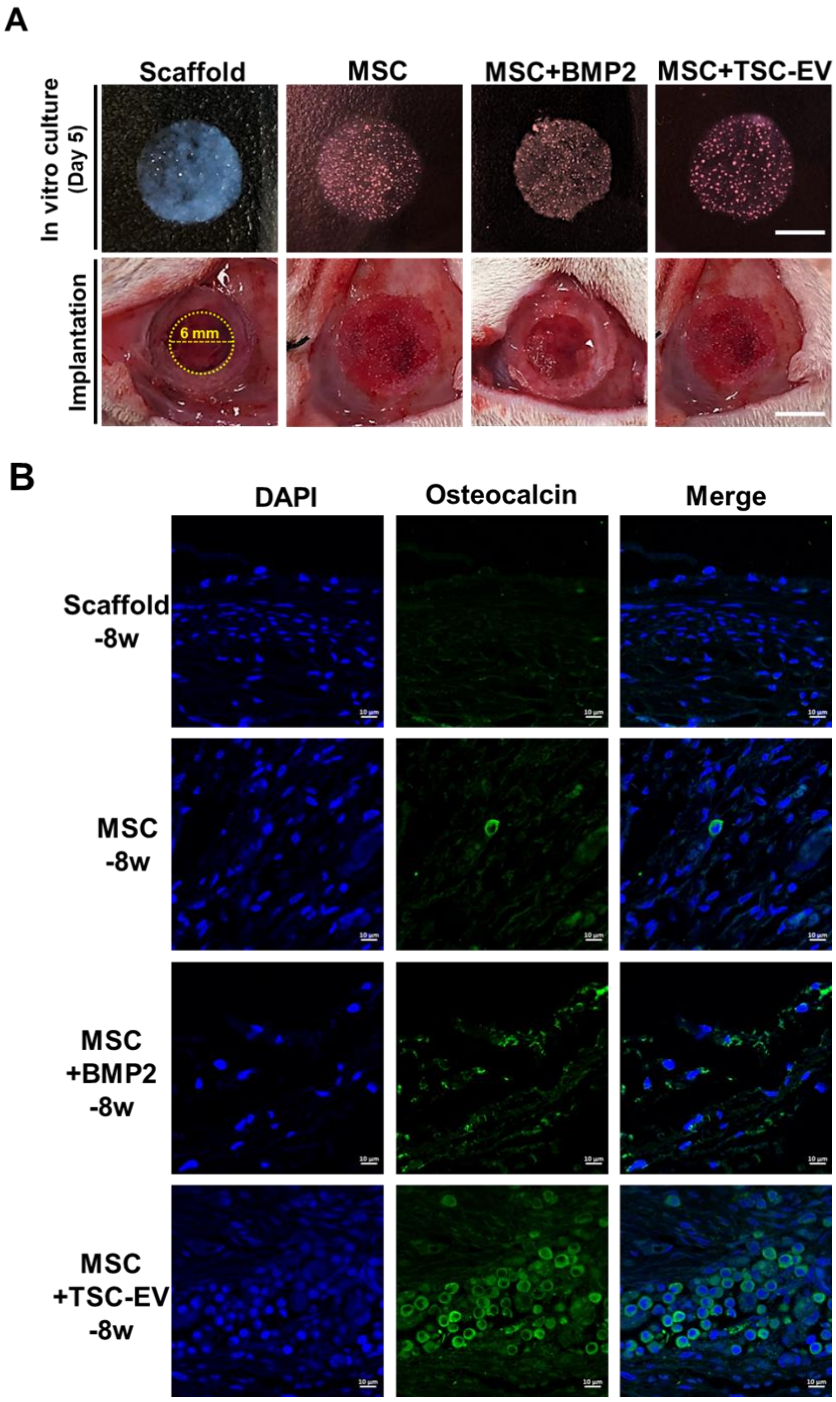

Supplementary Figure 6. Bone regeneration effect of TSC-EVs. (A) Representative images of scaffold, mesenchymal stem cell (MSC) only, MSC+BMP2, and MSC+TSC-EV groups after the 5 days of in vitro culture and implantation on calvaria bone defect. Scale bars, 0.5 mm. (B) Osteocalcin expression (green) was increased in TSC-EV treated MSC-laden group compared with remained groups. The immunofluorescence of DAPI (blue) and merge images are also showed here. Scale bars, 10 μm.

Supplementary Figure 7

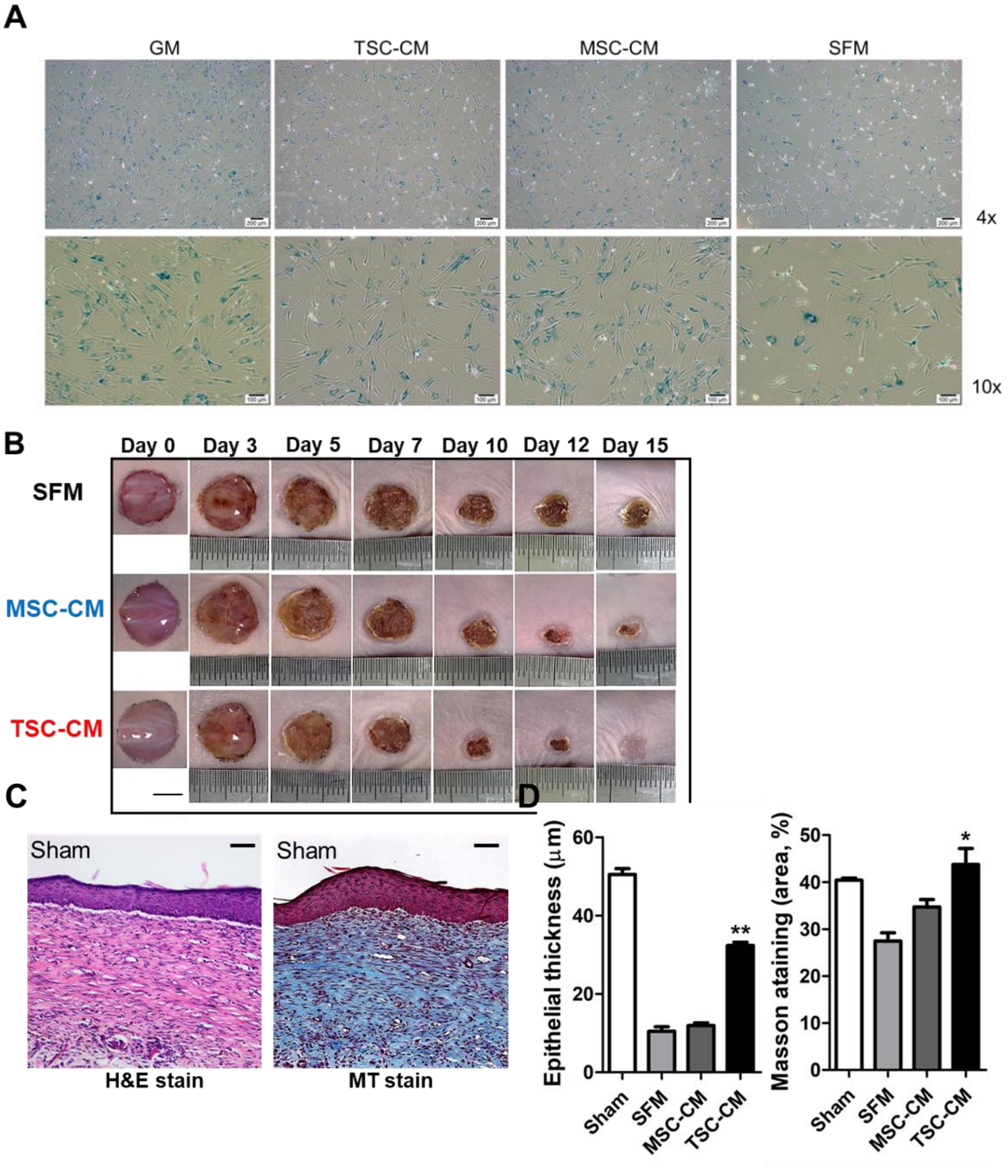

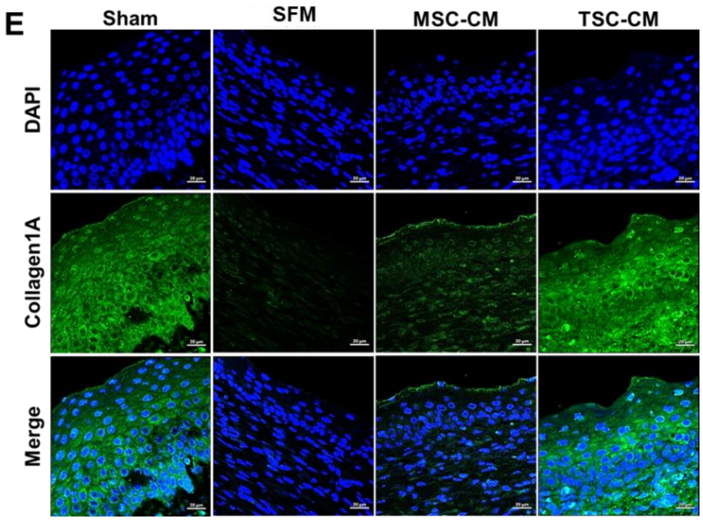

Supplementary Figure 7. Anti-senescence and wound-healing effects of TSC-derived secretomes. (A) A light microscopy images of SA-b-gal-stained MSCs cultured with GM, TSC-CM, MSC-CM, and SFM on different magnification; (n=3). Scale bars, 100 or 200 μm. (B) Representative photographs of wound-healing area in mice treated with SFM, MSC-CM and TSC-CM exposed MSCs on skin wound every 3 days postinjury; (n=6). Scale bars, 0.5 mm. (C) Hematoxylin and eosin (H&E) and Massion trichrome (MT) staining of skin area in mice (Sham); (n=4). (D) Quantitative analysis of H&E and MT staining compared with those of the sham group. Scale bars, 50 μm. Error bars presented the mean and SD. (*p<0.05 and **p<0.01) (E) Immunofluorescence images of collagen1A (green) in mice treated with SFM, MSC-CM and TSC-CM exposed MSCs on skin wound (DAPI, blue). Scale bars, 20 μm.

Supplementary Figure 8

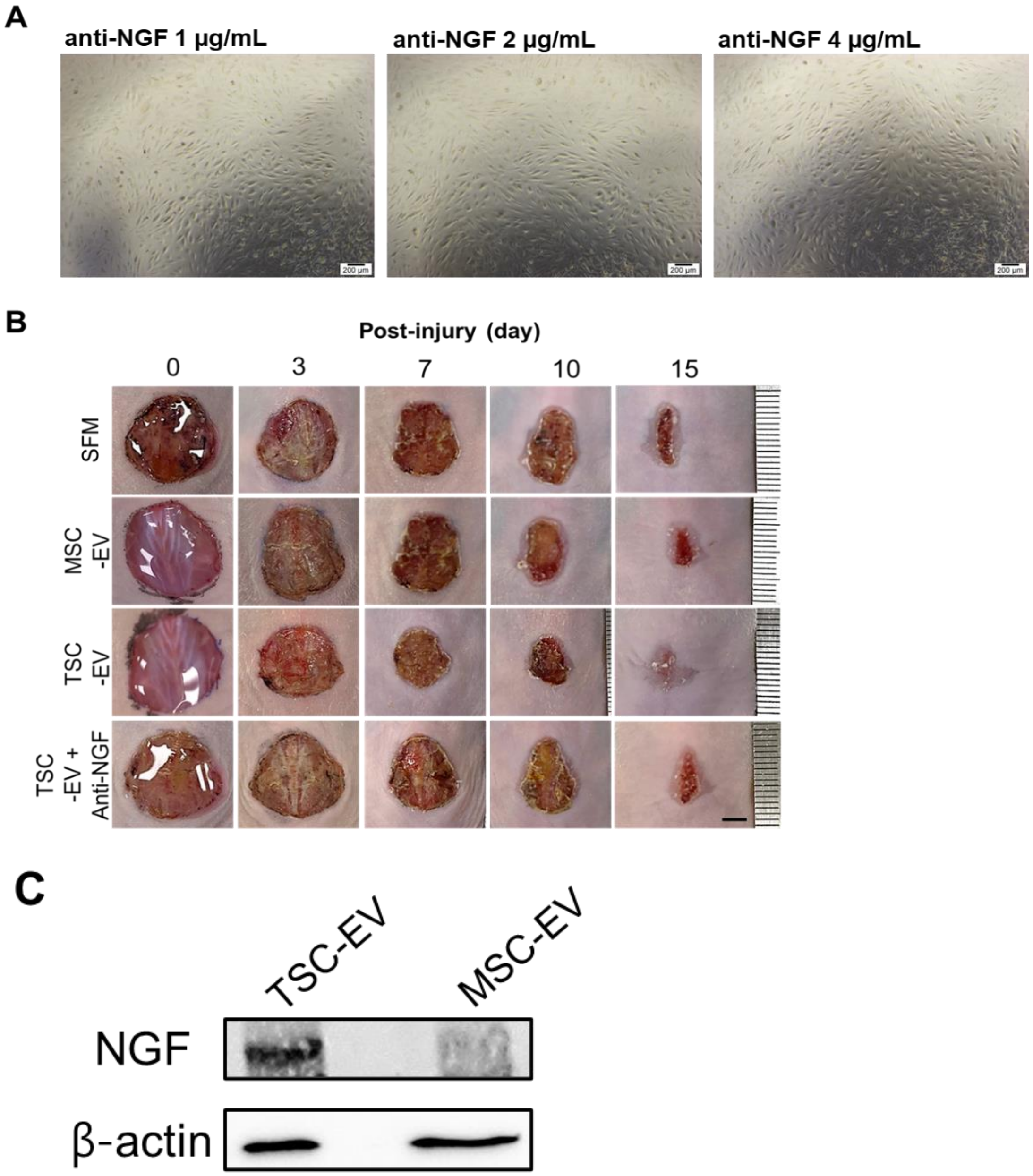

Supplementary Figure 8. Anti-NGF effect on MSCs and TSC-EV-treated MSCs. (A) Representative images in MSCs treated with anti-NGF 1, 2, and 4 ug/mL for 24 h. Scale bars, 200 µm. (B) Representative photographs of wound closure area in mice treated with SFM, MSC-EV, TSC-EV, TSC-EV+anti-NGF exposed MSCs on skin wound every 3 days postinjury; (n=6). Scale bars, 0.5 mm. (C) The protein levels of NGF in TSC- and MSC-EVs are compared by western blotting; (n=3).

Supplemental Table

Supplementary Table 1 Primer sequences used in qRT-PCR.

Gene	Primer Sequences
Human IBSP	F: 5’- GAACAAGGCATAAACGGCACC -3’<br>R: 5’- TTCTGCATTGGCTCCAGTGAC -3’
Human ALP	F: 5’- ACGAGCTGAACAGGAACAACGT -3’<br>R: 5’- CACCAGCAAGAAGAAGCCTTT -3’
Human RUNX2	F: 5’- GACACCACCAGGCCAATC -3’<br>R: 5’- AGAACAAGGGGGCCGTTA -3’
Human Osteix	F: 5’- GCCAGAAGCTGTGAAACCTC -3’<br>R: 5’- TGATGGGGTCATGGTGTCTA -3’
Human OCN	F: 5’- TGACGAGTTGGCTGACCA -3’<br>R:5’- AGGGTGCCTGGAGAGGAG -3’
Human OPN	F: 5’- AGGCATCACCTGTGCCATAC -3’<br>R: 5’- GATGGGTCAGGGTTTAGCCA -3’
Human OCT4	F: 5’- ATTCAGCCAAACGACCATC -3’<br>R: 5’- GGAAAGGGACCGAGGAGT -3’
Human NANOG	F: 5’- CAGCCCTGATTCTTCCACCAGTCCC -3’<br>R: 5’- TGGAAGGTTCCCAGTCGGGTTCACC -3’
Human SOX2	F: 5’- CAGCGCATGGACAGTTAC -3’<br>R: 5’- GGAGTGGGAGGAAGAGGT -3’
Human KLF4	F: 5’- GCGGGAAGGGAGAAGACA -3’<br>R: 5’- CCGGATCGGATAGGTGAA -3’
Human p53	F: 5’-ATCTACTGGGACGGAACAGC -3’<br>R: 5’-GTGAGGCTCCCCTTTCTTG -3’
Human NGFR	F: 5’-CGACAACCTCATCCCTGTCT-3’<br>R: 5’-TCGCTGTGGAGTTTTTCTCC-3’
Human GAPDH	F: 5’- TCGCCCCACTTGATTTTGG -3’<br>R: 5’- GCAAATTCCATGGCACCGT -3’

Supplementary Table 2. List of antibodies and chemical materials used in this study. * Western blotting (WB), Immunofluorescence (IF), neutralization (NE), Internalization (IN)

Materials	Source and identifier	Dilution	Application
Anti-Phospho-AKT antibody	Cell signaling (#9271S)	1:1,000	WB
Anti-AKT antibody	Cell signaling (#2922S)	1:1,000	WB
Anti-Collagen1A antibody	Santa Cruze (SC-59772)	1:500	IF
Anti-BrdU antibody	Invitrogen (B35130)	1:1,000	IF
DiR (Dil analog)	Invitrogen (D12731)	1:100	IN
Plasma membrane stain	Invitrogen (C10045)	1:1000	IF
Anti-OCN antibody	Santa Cruze (SC-390877)	1:500	IF
Anti-lamin A/C antibody	Abcam (ab 224816)	1:1000	IF
Anti-NGF antibody	R&D (MAB256-SP)	1:100	NE
Anti-NGF antibody	Peprotech (500-P85)	1:500	WB
Anti-CD81 antibody	SBI (EXOAB-CD81A-1)	1:1,000	WB
Anti-CD63 antibody	SBI (EXOAB-CD63-1)	1:1,000	WB
Anti-ALIX antibody	SBI (EXOAB-ALIX-1)	1:1,000	WB
Anti-beta Actin antibody	Santa Cruze (SC-47778)	1:2,000	WB
Anti-Rabbit IgG, HRP-linked antibody	Cell signaling (#7074S)	1:3,000	WB
Anti-Mouse IgG, HRP-linked antibody	Cell signaling (#7076S)	1:3,000	WB
Alexa Fluor ™ 488 donkey anti-rabbit IgG (H+L)	Invitrogen (A11008)	1:2,000	IF
Alexa Fluor ™ 488 goat a nti-mouse IgG (H+L)	Invitrogen (A11001)	1:2,000	IF
Alexa Fluor ™ 568 goat anti-mouse IgG (H+L)	Invitrogen (A11004)	1:2,000	IF

Supplementary Table 3 Statistics results of microRNA analysis data

Data	Total reads	Mapped reads	Known microRNA in sample	Known microRNA in species (miRbase v22.1)
MSCs	8,623,684	44,348 (0.51%)	294	2,656
TSCs	24,294,506	504,465 (2.08%)	331	2,656

Supplementary Excel Table 1. microRNA total read counts in TSC- and MSC-EV

	TSC-EV				MSC-EV		
	Gene Name	Read counts	Total read counts (%)		Gene Name	Read counts	Total read counts (%)
1	hsa-miR-1246	358225	71.61	1	hsa-let-7b-5p	7320	30.05
2	hsa-miR-1290	89371	17.87	2	hsa-miR-126-5p	3720	15.27
3	hsa-let-7b-5p	32281	6.45	3	hsa-miR-23a-3p	3134	12.87
4	hsa-miR-423-5p	8068	1.61	4	hsa-miR-23b-3p	1215	4.99
5	hsa-miR-335-5p	2823	0.56	5	hsa-miR-25-3p	767	3.15
6	hsa-let-7e-5p	1931	0.39	6	hsa-miR-4301	671	2.75
7	hsa-miR-574-5p	1206	0.24	7	hsa-miR-21-5p	612	2.51
8	hsa-miR-122-5p	781	0.16	8	hsa-let-7e-5p	543	2.23
9	hsa-let-7g-5p	780	0.16	9	hsa-miR-1246	521	2.14
10	hsa-let-7d-5p	721	0.14	10	hsa-let-7d-3p	416	1.71
11	hsa-miR-877-5p	620	0.12	11	hsa-miR-574-5p	414	1.70
12	hsa-miR-23a-3p	615	0.12	12	hsa-miR-150-5p	385	1.58
13	hsa-let-7i-5p	413	0.08	13	hsa-miR-92a-3p	347	1.42
14	hsa-miR-193b-5p	337	0.07	14	hsa-miR-423-5p	245	1.01
15	hsa-miR-98-5p	303	0.06	15	hsa-miR-486-5p	230	0.94
16	hsa-miR-130b-3p	138	0.03	16	hsa-let-7b-3p	230	0.94
17	hsa-miR-25-3p	134	0.03	17	hsa-miR-1290	195	0.80
18	hsa-miR-6126	111	0.02	18	hsa-miR-126-3p	183	0.75
19	hsa-miR-23b-3p	107	0.02	19	hsa-miR-142-3p	162	0.67
20	hsa-miR-21-5p	79	0.02	20	hsa-let-7a-3p	151	0.62
21	hsa-miR-320a-3p	74	0.01	21	hsa-miR-125a-5p	144	0.59
22	hsa-miR-130a-3p	69	0.01	22	hsa-miR-122-5p	133	0.55
23	hsa-miR-30b-3p	57	0.01	23	hsa-miR-223-3p	126	0.52
24	hsa-let-7d-3p	56	0.01	24	hsa-miR-199a-5p	125	0.51
25	hsa-miR-183-5p	56	0.01	25	hsa-miR-409-3p	121	0.50
26	hsa-miR-4525	50	0.01	26	hsa-miR-125b-5p	119	0.49
27	hsa-miR-378a-3p	47	0.01	27	hsa-let-7g-5p	102	0.42
28	hsa-miR-185-5p	40	0.01	28	hsa-miR-199a-3p	101	0.41
29	hsa-miR-1-3p	40	0.01	29	hsa-miR-199b-3p	101	0.41
30	hsa-miR-320b	39	0.01	30	hsa-miR-337-3p	93	0.38
31	hsa-miR-30c-2-3p	35	0.01	31	hsa-miR-221-3p	88	0.36
32	hsa-miR-125a-3p	34	0.01	32	hsa-let-7f-1-3p	82	0.34
33	hsa-miR-23a-5p	32	0.01	33	hsa-miR-222-3p	77	0.32
34	hsa-miR-625-5p	28	0.01	34	hsa-miR-1468-5p	75	0.31
35	hsa-miR-1910-3p	28	0.01	35	hsa-miR-142-5p	74	0.30
36	hsa-miR-935	25	0.00	36	hsa-miR-144-5p	67	0.28
37	hsa-miR-92b-5p	24	0.00	37	hsa-miR-130b-5p	62	0.25
38	hsa-miR-92a-3p	23	0.00	38	hsa-miR-144-3p	61	0.25
39	hsa-miR-30c-1-3p	19	0.00	39	hsa-miR-199b-5p	57	0.23
40	hsa-miR-32-3p	18	0.00	40	hsa-miR-98-5p	56	0.23
41	hsa-miR-664a-5p	18	0.00	41	hsa-miR-145-5p	54	0.22
42	hsa-miR-12136	17	0.00	42	hsa-miR-29b-3p	52	0.21
43	hsa-miR-619-5p	17	0.00	43	hsa-let-7d-5p	50	0.21
44	hsa-miR-296-3p	16	0.00	44	hsa-miR-451a	50	0.21
45	hsa-miR-744-5p	15	0.00	45	hsa-miR-6126	48	0.20
46	hsa-miR-126-5p	13	0.00	46	hsa-miR-214-3p	45	0.18
47	hsa-miR-197-5p	13	0.00	47	hsa-let-7i-5p	39	0.16
48	hsa-miR-4449	13	0.00	48	hsa-miR-1306-5p	38	0.16
49	hsa-miR-125a-5p	11	0.00	49	hsa-miR-4454	37	0.15
50	hsa-miR-320c	11	0.00	50	hsa-miR-143-3p	34	0.14
51	hsa-miR-766-5p	11	0.00	51	hsa-miR-365a-3p	33	0.14
52	hsa-miR-378c	10	0.00	52	hsa-miR-365b-3p	33	0.14
53	hsa-miR-483-5p	10	0.00	53	hsa-miR-186-5p	28	0.11
54	hsa-miR-5584-5p	10	0.00	54	hsa-miR-7-1-3p	26	0.11
55	hsa-miR-6788-5p	10	0.00	55	hsa-miR-101-3p	25	0.10
56	hsa-miR-6885-5p	10	0.00	56	hsa-miR-1260b	24	0.10
57	hsa-miR-9-3p	10	0.00	57	hsa-miR-411-3p	23	0.09
58	hsa-let-7a-3p	9	0.00	58	hsa-miR-660-5p	22	0.09
59	hsa-miR-3126-5p	9	0.00	59	hsa-miR-193b-5p	18	0.07
60	hsa-miR-3138	9	0.00	60	hsa-miR-379-3p	17	0.07
61	hsa-miR-320d	9	0.00	61	hsa-miR-34a-5p	16	0.07
62	hsa-miR-342-5p	9	0.00	62	hsa-miR-22-3p	16	0.07
63	hsa-miR-222-3p	8	0.00	63	hsa-miR-130a-3p	15	0.06
64	hsa-miR-1224-5p	8	0.00	64	hsa-miR-369-3p	14	0.06
65	hsa-miR-29b-3p	7	0.00	65	hsa-miR-329-3p	14	0.06
66	hsa-miR-149-3p	7	0.00	66	hsa-miR-664a-3p	14	0.06
67	hsa-miR-1908-5p	7	0.00	67	hsa-miR-181a-5p	13	0.05
68	hsa-miR-3679-5p	7	0.00	68	hsa-miR-425-5p	13	0.05
69	hsa-miR-378d	7	0.00	69	hsa-miR-122-3p	13	0.05
70	hsa-miR-378g	7	0.00	70	hsa-miR-1260a	13	0.05
71	hsa-miR-125b-5p	6	0.00	71	hsa-miR-26a-2-3p	13	0.05
72	hsa-miR-6794-5p	6	0.00	72	hsa-miR-34a-3p	12	0.05
73	hsa-miR-765	6	0.00	73	hsa-miR-380-3p	12	0.05
74	hsa-miR-130b-5p	5	0.00	74	hsa-miR-483-3p	12	0.05
75	hsa-miR-486-5p	4	0.00	75	hsa-miR-877-5p	11	0.05
76	hsa-miR-409-3p	4	0.00	76	hsa-miR-27a-3p	11	0.05
77	hsa-miR-221-3p	4	0.00	77	hsa-miR-379-5p	11	0.05
78	hsa-let-7f-1-3p	4	0.00	78	hsa-miR-495-3p	11	0.05
79	hsa-miR-143-3p	4	0.00	79	hsa-miR-136-3p	10	0.04
80	hsa-miR-186-5p	3	0.00	80	hsa-miR-382-3p	9	0.04
81	hsa-let-7b-3p	2	0.00	81	hsa-miR-409-5p	9	0.04
82	hsa-miR-142-5p	2	0.00	82	hsa-miR-194-5p	8	0.03
83	hsa-miR-214-3p	2	0.00	83	hsa-miR-340-3p	8	0.03
84	hsa-miR-411-3p	2	0.00	84	hsa-miR-487a-5p	8	0.03
85	hsa-miR-4301	1	0.00	85	hsa-miR-532-3p	8	0.03
86	hsa-miR-126-3p	1	0.00	86	hsa-miR-6529-3p	8	0.03
87	hsa-miR-223-3p	1	0.00	87	hsa-miR-378a-3p	7	0.03
88	hsa-miR-1468-5p	1	0.00	88	hsa-miR-335-5p	6	0.02
89	hsa-miR-199b-5p	1	0.00	89	hsa-miR-130b-3p	6	0.02
90	hsa-miR-145-5p	1	0.00	90	hsa-miR-185-5p	6	0.02
91	hsa-miR-4454	1	0.00	91	hsa-miR-23a-5p	3	0.01
92	hsa-miR-7-1-3p	1	0.00	92	hsa-miR-125a-3p	2	0.01
93	hsa-miR-101-3p	1	0.00	93	hsa-miR-625-5p	2	0.01
94	hsa-miR-34a-5p	1	0.00	94	hsa-miR-30c-1-3p	2	0.01
95	hsa-miR-369-3p	1	0.00	95	hsa-miR-12136	2	0.01
96	hsa-miR-181a-5p	1	0.00	96	hsa-miR-320a-3p	0	0.00
97	hsa-miR-425-5p	1	0.00	97	hsa-miR-30b-3p	0	0.00

	TSC-Exos	MSC-Exos
Total read counts	500,223	24,359
Fold change (TSC/MSC)		
20.53544891		

Source Data of Western Blotting

Figure 7B

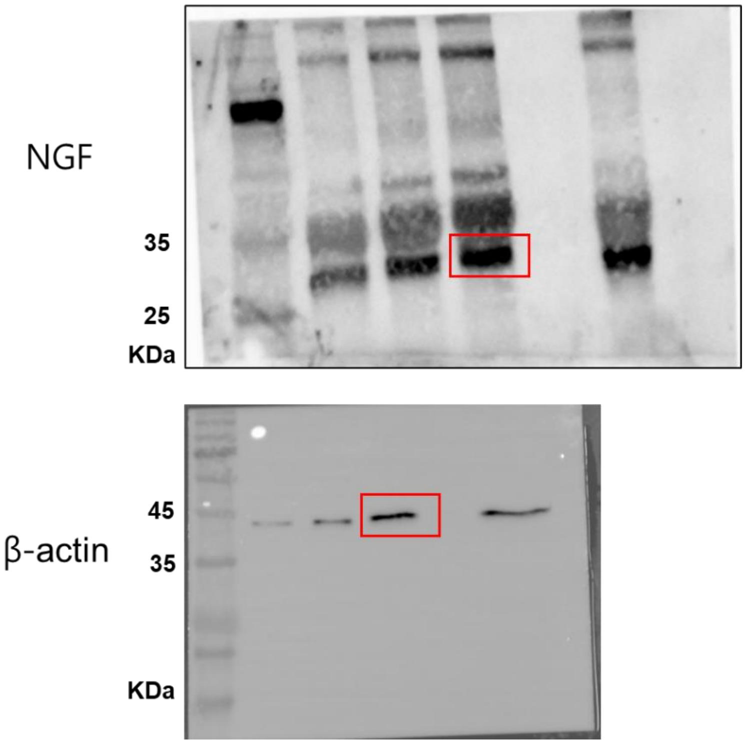

Figure 7C

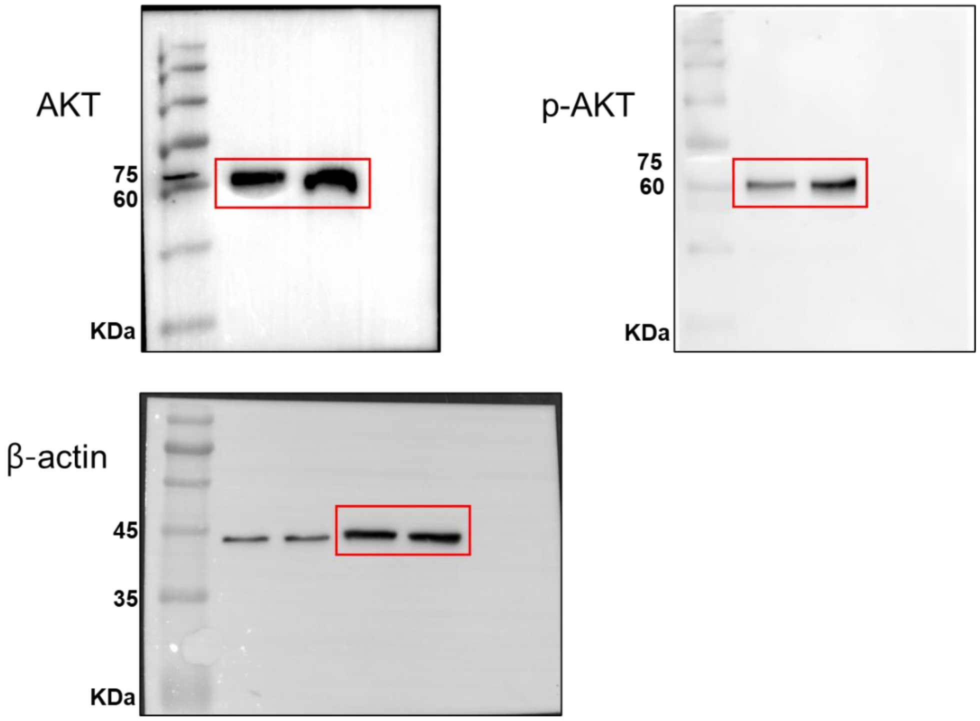

Figure 7E

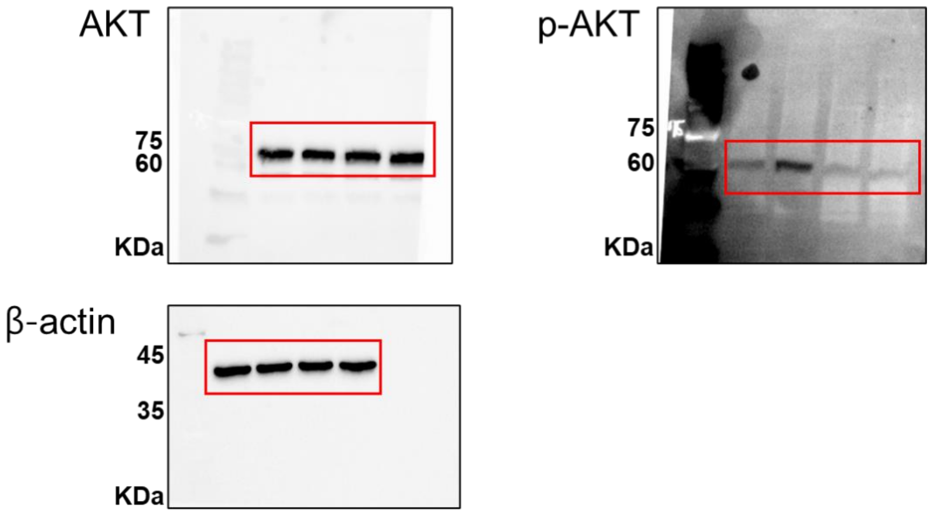

Supplementary Figure 1D

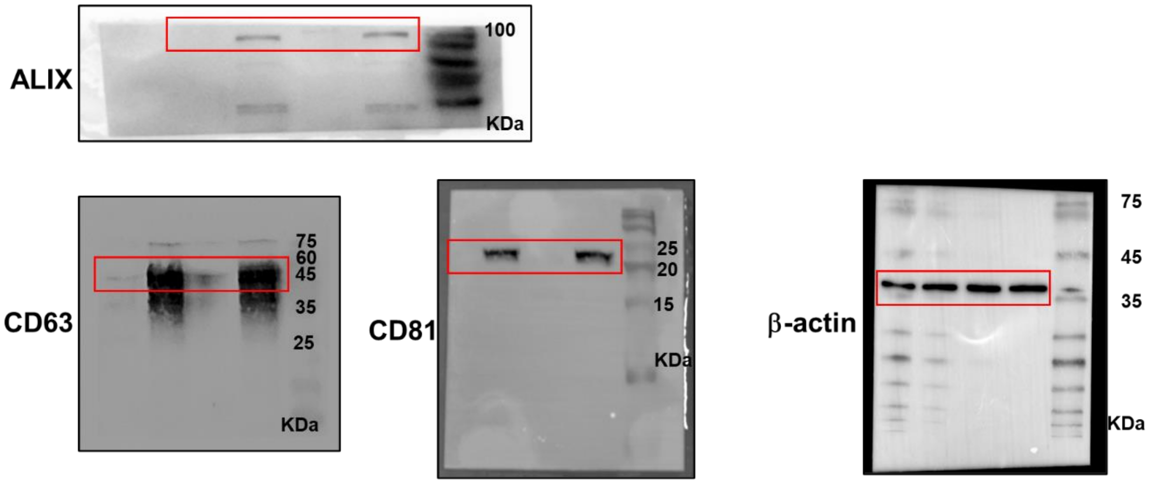

Supplement: Supplementary file 1 — Additional file 1: Supplemental Methods. Supplementary Figure 1. Flow chart of transcriptomic analysis in this study. Supplementary Figure 2. Characterization of TSC-EV and MSC-EV. Supplementary Figure 3. Increased proliferation rate of human MSCs via TSC-derived CM and EV. Supplementary Figure 4. Significant biological processes and pathway of four microRNAs primed by TSC-EVs. Supplementary Figure 5. DEGs in significant KEGG pathways and enriched GO terms in TSC-EV-treated MSCs. Supplementary Figure 6. Bone regeneration effect of TSC-EVs. Supplementary Figure 7. Anti-senescence and wound-healing effects of TSC-derived secretomes. Supplementary Figure 8. Anti-NGF effect on MSCs and TSC-EV-treated MSCs. Supplementary Table 1. Primer sequences used in qRT-PCR. Supplementary Table 2. List of antibodies and chemical materials used in this study. Supplementary Table 3. Statistics results of microRNA analysis data. Supplementary Excel Table 1. microRNA total read counts in TSC- and MSC-EV. Source Data of Western Blotting. [file 40824_2023_396_MOESM1_ESM.zip › Supplementary Figures_ESM.pdf]
